# A chromatin scaffold for DNA damage recognition: how histone methyltransferases prime nucleosomes for repair of ultraviolet light-induced lesions

**DOI:** 10.1093/nar/gkz1229

**Published:** 2020-01-13

**Authors:** Corina Gsell, Holger Richly, Frédéric Coin, Hanspeter Naegeli

**Affiliations:** 1 Institute of Pharmacology and Toxicology, University of Zurich-Vetsuisse, Winterthurerstrasse 260, 8057 Zurich, Switzerland; 2 Boehringer Ingelheim Pharma, Department of Molecular Biology, Birkendorfer Str. 65, 88397 Biberach an der Riß, Germany; 3 Institut de Génétique et de Biologie Moléculaire et Cellulaire, Equipe Labélisée Ligue contre le Cancer, Illkirch Cedex, Strasbourg, France

## Abstract

The excision of mutagenic DNA adducts by the nucleotide excision repair (NER) pathway is essential for genome stability, which is key to avoiding genetic diseases, premature aging, cancer and neurologic disorders. Due to the need to process an extraordinarily high damage density embedded in the nucleosome landscape of chromatin, NER activity provides a unique functional caliper to understand how histone modifiers modulate DNA damage responses. At least three distinct lysine methyltransferases (KMTs) targeting histones have been shown to facilitate the detection of ultraviolet (UV) light-induced DNA lesions in the difficult to access DNA wrapped around histones in nucleosomes. By methylating core histones, these KMTs generate docking sites for DNA damage recognition factors before the chromatin structure is ultimately relaxed and the offending lesions are effectively excised. In view of their function in priming nucleosomes for DNA repair, mutations of genes coding for these KMTs are expected to cause the accumulation of DNA damage promoting cancer and other chronic diseases. Research on the question of how KMTs modulate DNA repair might pave the way to the development of pharmacologic agents for novel therapeutic strategies.

## INTRODUCTION

Genome stability is constantly threatened by endogenous and exogenous DNA-damaging agents that induce a variety of DNA base or backbone lesions. A network of DNA repair processes avoids the conversion of DNA damage to mutations and chromosomal aberrations, thus preventing genetic diseases, premature aging, cancer and other chronic conditions (1–3). Nucleotide excision repair (NER) is the DNA repair process dedicated to the removal of DNA adducts that are typically larger than normal nucleotides. Prominent examples of such bulky lesions, arising at high frequency in the genome, are di-pyrimidine crosslinks induced by ultraviolet (UV) light and DNA adducts generated by chemical carcinogens. In particular, UV radiation is the most common environmental genotoxic agent and the major etiological factor for the development of skin cancer. Depending on the locality, season, time of day, weather conditions and the period of exposure, an assault by the short-wave sunlight spectrum generates in each skin cell hundreds of thousands of covalent crosslinks between neighboring pyrimidines, predominantly cyclobutane pyrimidine dimers (CPDs) and pyrimidine–pyrimidone (6-4) photoproducts (6–4PPs) in a stoichiometry of ∼4:1 (4–6). The multipronged cellular responses to this genotoxic insult can only be understood through analyses in the physiologic context of the tightly packed chromatin substrate.

## DISTRIBUTION OF DNA DAMAGE IN CHROMATIN

A compaction of DNA in the nuclei of eukaryotic cells is imposed by the need to package the genome (consisting of ∼6.4 × 10^9^ bp in a diploid human cell) into its tight nuclear compartment. To remain accessible for genomic functions, DNA filaments are assembled with histones to form a condensed but highly adjustable supramolecular array whose repeating unit is the nucleosome. Each nucleosome repeat comprises a core particle consisting of 147 bp of the DNA helix wrapped ∼1.7 times around a histone H3–H4 tetramer flanked by two H2A–H2B dimers. These core particles are intrinsically stable due to the many electrostatic contacts between the negatively charged DNA backbone and positively charged residues of the basic histones. Linker DNA of variable length (20–50 bp) connects core particles and, in higher eukaryotes, histone H1 induces additional compaction (7–9). This ‘chromatinization’ with ∼30 million nucleosomes per diploid human genome does not generally avert genotoxic reactions, although it modulates their incidence, but restricts the accessibility for subsequent DNA repair reactions. As an example, the induction of CPDs and resulting mutations are modulated by transcription factors and histones (10,11). Throughout chromatin, however, these lesions occur rather uniformly (12–14). There is only a small (maximally twofold) bias for CPD formation in linker DNA with the consequence that most UV lesions in a damaged genome are found inside the 147 bp of nucleosome cores (14–16). How NER factors detect DNA lesions in this ‘chromatinized’ substrate is a long-standing question that has been the focus of intense research. Repair assays in reconstituted cell-free systems indicated that the assembly of DNA into nucleosomes is, in principle, inhibitory to UV lesion excision (17–20). However, UV damage repair is efficient in intact living cells, implying that chromatin is temporarily rearranged to allow for NER activity (21,22).

## THE ACCESS–REPAIR–RESTORE MODEL FOR NER ACTIVITY IN CHROMATIN

The cut-and-patch NER reaction has been elucidated in detail (23–25), but the mechanism allowing for bulky lesion recognition in the chromatin context remains to be understood. Because DNA damage in chromatin is refractory to repair, NER activity requires that nucleosomes are mobilized by ATP-dependent chromatin remodelers and posttranslational histone modifications (21,26–28). A powerful tool for the spatiotemporal analysis of chromatin rearrangements came from DNA dissection by micrococcal nuclease (MNase). This enzyme digests DNA more easily in the accessible linker segments than that in histone-assembled nucleosome cores. Therefore, MNase generates a soluble supernatant containing proteins that, before digestion, were associated with linker DNA as well as a minor fraction of isolated core particles referred to as ‘mono-nucleosomes’. Even at saturating enzyme levels, this MNase digestion leaves behind the vast majority of core particles (containing DNA fragments of 147 bp) in the form of a densely packed and insoluble nucleoprotein fraction (29). In a pioneering experiment, this enzyme was used to monitor the fate of excision repair patches in UV-exposed fibroblasts. The cells were pulse-labeled with radioactive thymidine immediately after irradiation and, then, chased in non-radioactive medium for different times. Smerdon and Liebermann (30) observed that, initially, most of the incorporated radioactivity reflecting repair synthesis occurs in MNase-sensitive chromatin although, with increasing chase time, this radioactivity becomes progressively nuclease-resistant. This finding implies that DNA repair patches are not synthesized within nucleosomes but, instead, are subjected to nucleosome packaging after completion of the repair reaction. Such rearrangements involving chromatin disassembly and reassembly have later been confirmed by genome-wide analyses of nucleosome occupancy, based on high-resolution mapping by DNA sequencing (31), and gave rise to the ‘access, repair, restore’ model to account for the efficient excision of CPDs formed in nucleosome cores (27,28). It is not yet clear if chromatin rearrangements during UV lesion repair involve nucleosome sliding, destabilization or disruption (32). In any case, DNA repair synthesis is accompanied by chromatin restoration through nucleosome repositioning mediated by histone chaperones (33,34). Histones are escorted during DNA repair synthesis by the chaperone CAF-1 (Chromatin Assembly Factor-1), whose largest subunit interacts with the DNA polymerase sliding clamp PCNA (Proliferating Cell Nuclear Antigen) (35–37). CAF-1 interacts with another histone chaperone known as ASF1 (Anti-Silencing Function 1) (38) that serves as the histone donor (39). This chromatin restoration process also involves the deposition of the histone variants H2.X and H3.3 by the histone chaperones FACT (FAcilitates Chromatin Transcription) and HIRA (HIstone Regulator A), respectively (40–42).

## DOES CHROMATIN STABILIZATION PRECEDE DESTABILIZATION?

The ‘access-repair-restore’ model, outlined above, proposes that the chromatin structure must be disrupted or at least weakened to expose damaged sites to DNA repair factors. This model raises the fundamental question of whether chromatin relaxation precedes lesion detection or *vice versa*. A possible solution to this conundrum is that chromatin should, perhaps, not be considered as an absolute barrier to repair processes in a way that nucleosomes need to be dissolved for the initial access of recognition factors to DNA lesions. Instead, several counterintuitive findings lend support to the view that chromatin may have a scaffolding role to facilitate DNA damage recognition.

One important notion is that the pattern of inflicted damage follows the bending of DNA around the surface of the histone octamer in a way that nucleosomes modulate UV lesion formation by favoring the more accessible outside facing sites and disfavoring less accessible inward sites in the proximity to histones (43,44). A more intriguing observation is that heterochromatin protein 1 (HP1) family members are recruited to various DNA damages, including UV lesions, independently of DNA repair (45). Proteins of the HP1 family (HP1α, HP1β, HP1γ) localize to contracted heterochromatin regions like centromeres and telomers, but rapidly switch between free and chromatin-bound states (46). The loss of HP1 isoforms is lethal in mammalian cells, but a function of these chromatin-condensing factors in the DNA damage response has been demonstrated in *Caenorhabditis elegans*, as mutant nematodes missing the two *C. elegans* isoforms HPL-1 and HPL-2 are highly UV radiation-sensitive (45). Another surprising observation is that an accumulation of the aforementioned histone chaperone HIRA at UV-damaged sites takes place already during early NER steps before incision/excision/repair synthesis and, in fact, this HIRA recruitment depends on the cullin 4-RING ubiquitin ligase (CRL4) activity that accompanies the initial lesion recognition (see below). The transient HIRA enrichment in damaged chromatin is followed by an increased deposition of the histone H3.3 variant before execution of the NER reaction (47). Notably, the function of HP1 and HIRA in the DNA damage response is not limited to the UV response but extends to other types of lesions including DNA double strand breaks (DSBs) (47,48). Another unexpected finding came from chromatin immunoprecipitation (ChIP) experiments using XPC protein as the bait. The precipitated nucleoprotein complexes contain histone H3, unmodified and trimethylated at position lysine 9 (H3K9me3), but are essentially devoid of some acetylated forms of histone H3. Similarly, the induction of UV lesions in the nuclei of human cells, by irradiation through 5 μm-wide pores, generated subnuclear spots of UV damage that are depleted of acetylated histone H3 (49). These observations are surprising because acetylation is a posttranslational histone modification that increases the accessibility of DNA-binding factors and, in fact, the presence of acetylated histones correlates with the relaxed chromatin of transcribed genes (50). These findings indicate that histones are transiently deacetylated in response to UV damage and, consequently, that NER activity is not regulated in the same way as transcription.

Taken together, these reports describing the recruitment of HP1 and HIRA, the latter promoting histone deposition, as well as the loss of histone acetylation in response to UV damage, suggest that the immediate DNA damage response involves a stabilization of the nucleosome structure. This conclusion is supported by studies with cells of the silkworm *Bombyx mori*, revealing that polycomb repressing complexes (PRCs), which lead to chromatin compaction, also participate in the UV response (51). Why do proteins that promote a condensed chromatin state accompany early NER reactions? A possible explanation is that a transient chromatin contraction upon DNA insults serves to block transcription and avoid molecular collisions between RNA polymerases and repair enzymes (52). In keeping with this hypothesis, another report showed that, during DSB repair, transcription is switched off by recruiting PRC1 via ATM-mediated phosphorylation (53). In the case of UV lesions, however, we favor the alternative explanation that safeguarding the stability of nucleosomes around injured sites is critical for DNA damage recognition and initiation of NER activity. This mechanism may counteract the nucleosome-destabilizing effect of DNA damage, thus avoiding a premature histone eviction in view that, for example, a single CPD lesion is sufficient to induce unwrapping of the nucleosome core (54,55). We propose that nucleosome arrays provide an indispensable scaffold for damage recognition during NER activity and that, in this context, certain KMTs introduce a histone code that makes this scaffold more permissive to NER transactions.

## MAMMALIAN CORE NER REACTION

Throughout evolution, the NER machine is the only available toolbox for the removal of bulky lesions. Its cut-and-patch reaction course involves the excision of DNA adducts by dual incision of damaged strands, followed by their release as part of 24- to 32-nucleotide long single-stranded segments (56,57). This repair process is initiated by two alternative recognition modes. In transcription-coupled repair (TCR), damage excision is triggered by collision of the elongating RNA polymerase II (RNA Pol II) with DNA lesions and this roadblock prioritizes the excision of damage located on transcribed strands. By backtracking of the stalled RNA Pol II and promoting the repair of transcribed strands, the TCR pathway allows for a rapid recovery of transcription to prevent apoptosis (58–60). Instead, the genome-wide NER activity, which includes the repair of non-transcribed strands, is termed global-genome repair (GGR) (3,61). This GGR pathway prevents replication forks from encountering DNA lesions, thus mitigating damage-induced mutagenesis. Genetic GGR defects result in xeroderma pigmentosum (XP), a cancer-prone syndrome presenting with photosensitivity, severe sunburns, skin photoaging, ocular pathologies and, in the absence of sunlight protection, a >1000-fold increased risk of skin cancer. GGR defects also confer a higher susceptibility to lung, breast and colorectal cancer, and XP patients also suffer from neurologic disorders (25,62–64).

**Figure 1.**
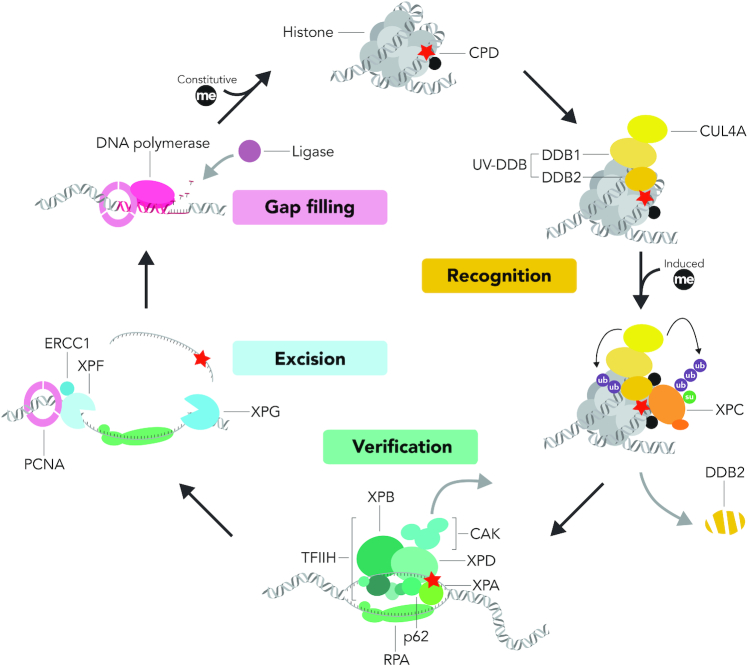
GG-NER reaction in chromatin. This scheme summarizes the major transitions of the GGR reaction cycle (from DNA damage recognition to the final DNA gap filling) and the ‘access-repair-restore’ model describing how this multistep process may take place in the nucleosome landscape of chromatin. The XPC subunit initiates GGR activity as part of a trimeric complex with HR23B and CETN2. KMTs favor the GGR reaction in the nucleosome context by constitutive and DNA damage-induced depositions of histone methylation marks.

Mechanistically, the GGR system uses a complex consisting of XPC protein, one of two RAD23 homologs (primarily RAD23B) and centrin 2 (CETN2) (65–68) to detect bulky lesions anywhere in the genome (Figure 1). The XPC subunit functions as a sensor of base pairs with reduced Watson–Crick stability compared to the native base complementarity. This indirect recognition mode, by which XPC detects unpaired but intact bases in the undamaged strand, explains how the GGR reaction achieves a wide substrate range despite the absence of a common chemical structure of the offending adducts (69–72). It has been estimated that a human cell may contain between 25 000 (73) and 80 000 XPC molecules (74) such that, considering the size of the diploid human genome, each XPC molecule would need to interrogate up to 250 000 bp. Fluorescence-based protein dynamics studies showed that a DNA-repulsive motif of XPC protein, favoring dissociation of this DNA-binding subunit from native DNA, is important to achieve the high mobility required for an effective target search (67,75). In line with this observation, quantum dot-based tracking experiments revealed that the XPC–HR23B complex diffuses along the DNA helix by a ‘hopping’ mechanism *via* repeated dissociation and nearby re-association events (76). In this ‘hopping’ scenario of protein movement, nucleosomes could be either an obstacle or provide a grip for XPC protein and, accordingly, histone modifiers are likely to play a crucial role for the target search.

Once bound to DNA lesion sites, RAD23B dissociates and the remaining XPC-CETN2 dimer forms a landing platform for the 10-subunit TFIIH (Transcription Factor IIH) complex to allow for damage verification (77–79). This recruitment is mediated by interactions of XPC protein with the p62 and XPB subunits of TFIIH together with the use of the ATPase activity of XPB to anchor the complex onto the DNA substrate (80–82). Subsequently, the TCR and GGR reactions converge on a joint pathway where the recruitment of XPA triggers the release of the CAK (CDK-Activating Kinase) sub-complex of TFIIH and launches the XPD helicase subunit of TFIIH, which unwinds the double helix and thereby scans the targeted DNA strand for abnormal chemical structures (83–86). The XPD-mediated unwinding extends over ∼25 nucleotides (87,88) and generates a ‘bubble’ intermediate that is stabilized by RPA (Replication Protein A) together with XPA (89,90), and these two factors assemble a DNA incision machinery. The damaged strand is in that way cleaved at the double- to single-stranded DNA junctions on each side of the ‘bubble’. A heterodimer of XPF and ERCC1 (Excision Repair Cross-Complementing 1) makes the 5′ cut, followed by incision on the 3′ side through XPG (91). After this dual incision and release of an oligonucleotide carrying the lesion, integrity of the double helix is restored by DNA repair synthesis (92) and ligation (73,93).

## PYRIMIDINE DIMER COMPLEXITIES

The two major di-pyrimidine lesions induced by sunlight differ with regard to structure and biological effects. First, CPDs occur more abundant than 6–4PPs. Second CPDs cause a minor thermodynamic destabilization of Watson–Crick base pairs compared to 6–4PPs (94–96) such that, without further assistance, the XPC initiator of GGR activity would fail in their detection. Instead, XPC protein is able to sense on its own the more pronounced base pair destabilization caused by 6–4PPs (97,98). Third, there is as mentioned before a rather uniform distribution of CPDs in the genome, whereas 6–4PPs are found predominantly in linker DNA (43,99,100). Fourth, CPDs are excised at slower rates than 6–4PPs and, consequently, are responsible for the adverse UV radiation effects including sunburns, skin photoaging and cutaneous cancer (101–103).

The detection of CPDs generally depends on an auxiliary DNA damage recognition complex known as UV-DDB (UV-damaged DNA-binding; Figure 1). The DDB2 subunit of this heterodimer acts as a UV lesion receptor that inserts itself like a wedge into the damaged double helix and interacts directly with the lesion by accommodating the pyrimidine dimer into a shallow binding pocket (104–107). By connecting with its adaptor DDB1, this direct recognition of UV lesions leads to the recruitment of the cullin 4A scaffold and the RING finger protein ROC1, which build the CRL4^DDB2^ ubiquitin ligase that modifies DDB2 itself, XPC protein (108) and histones (109,110) with ubiquitin moieties. K48-linked ubiquitin chains trigger the proteasomal degradation of DDB2 after extraction from chromatin by the ubiquitin-dependent p97 segregase (111,112) to allow for follow-up GGR events (113–115). Ubiquitinated XPC protein is also extracted by the p97 segregase, but without degradation, and a further role of ubiquitin chains in regulating the spatial protein distribution is discussed below. It should be mentioned that XPC undergoes another round of ubiquitination via the RNF111 ubiquitin ligase (also known as Arkadia), which attaches K63-linked ubiquitin chains (116,117). This extra ubiquitination by Arkadia is dependent on the prior modification of XPC protein with SUMO (Small Ubiquitin-related MOdifier), which has been implicated in promoting a rapid release of UV-DDB from its association with the XPC complex (118). These modifications of DDB2 and XPC are, therefore, indispensable for the well-timed substrate handover from one factor to the next along the GGR pathway. With regard to histones, it has been proposed that the CRL4^DDB2^-mediated ubiquitination of H2A, H3 and H4 in response to UV radiation helps opening chromatin, thus facilitating access of downstream GGR players after the initial lesion detection (109,110,119). It is also known that UV-DDB recruits chromatin remodelers (26), the histone chaperone HIRA (47), PARP1 [Poly(ADP-Ribose)Polymerase 1] (120,121) and histone modifiers like acetyltransferases (122–125), which may all contribute to rearranging chromatin for CPD repair.

## NUCLEOSOMES AS A SCAFFOLD FOR GGR INITIATION

The predominant occurrence of CPDs within nucleosome cores raises the question of whether the tight wrapping of DNA around histones might render these lesions more or less recognizable. Interestingly, the crystal structure of a core particle containing CPD lesions revealed that, unlike their nearly native Watson-Crick configuration in the naked double helix, the two crosslinked pyrimidines do not form regular hydrogen bonds, i.e. one pyrimidine is displaced away from its complementary purine, indicating that Watson–Crick base pairing is substantially destabilized (126). In view of this eye-catching feature of a base pair destabilization provoked by coiling the DNA around histone octamers, the CPD lesion might become more conducive to recognition by both UV-DDB, the UV lesion receptor, and XPC protein, the GGR initiator. Such considerations may have a broader relevance for carcinogen-DNA adducts in general. In fact, molecular dynamics studies suggest that histone tails might favor the base pair-disrupted conformation induced by a *cis*-benzo[*a*]pyrene-dG adduct positioned within a nucleosome core particle, perhaps further exposing the flipped-out base to promote recognition by XPC protein (127).

Irrespective of the detailed structural characteristics of damaged nucleosomes, UV-DDB has been shown to associate tightly with chromatin following UV irradiation (128) and to remain bound to mono-nucleosomes when the chromatin of UV-irradiated cells is solubilized by MNase digestion (129). That UV-DDB is able to detect CPDs in chromatin was demonstrated by reconstituting nucleosome core particles with DNA fragments, containing a site-directed CPD, and recombinant human core histones assembled into octamers. Electrophoretic mobility shift assays (EMSAs) unequivocally demonstrated that UV-DDB binds to the site-specific CPD even when this lesion is incorporated into the reconstituted core particles (105). Cryo-electron microscopy studies indicate that DDB2, the damage receptor subunit of UV-DDB, avoids clashes with histone residues while binding to UV lesions in nucleosomes. In the case of nucleosome-buried lesions that face the inside histone core, UV-DDB shifts the position of DNA relative to the histone octamer in a way that the lesion reaches a more outward localization facing away from the histone core (32). In contrast to these findings obtained with UV-DDB, inconclusive results were generated from studies carried out to test the binding of XPC protein to nucleosomes. The above described *in vitro* assembly of core particles, with a site-directed UV lesion, suppressed the binding of the XPC-HR23B complex to the lesion site as measured by EMSA (130). Another report showed that XPC protein binds to nucleosome core particles *in vitro* without dissociating the histone-DNA complex, although only at high concentrations and without being able to discriminate between damaged and undamaged substrates (18). These findings could be taken as evidence that XPC protein, on its own, is unable to detect damaged DNA sites within nucleosome cores.

Protein dynamics studies based on fluorescent fusion tags, combined with fluorescence recovery after local photobleaching in living cells, revealed a low nuclear mobility of XPC protein, indicating that this factor, in contrast to other NER subunits, is constantly associated with chromatin fibers (131). Additionally, electron microscopy analysis showed that the XPC complex occupies condensed chromatin regions whereas all the other NER factors are recruited preferentially to UV lesions in less condensed chromatin regions (132). Accordingly, XPC protein also colocalizes with mitotic chromosomes (74,131). The presence of XPC protein in compacted chromatin has been attributed to the affinity of this factor for DNA in general and the higher DNA concentration in condensed chromatin. In view of the long-held notion, derived from *in vitro* reconstitution experiments (18,130), that nucleosomes pose a barrier to the binding of XPC protein to the DNA substrate, it was very surprising to find upon MNase digestion of chromatin that, instead, a substantial fraction of XPC protein associates with the nuclease-resistant fraction rich in core particles (133). The proportion of XPC protein associated with core particles even increases upon UV irradiation, suggesting that, in intact cells, the XPC complex is able to detect UV lesion sites even when the damaged DNA is wrapped around histones. Further chromatin fractionation experiments, carried out under conditions that resulted in inhibition of CRL4^DDB2^ activity, showed that the UV-DDB-dependent ubiquitination of the XPC subunit serves to retain this factor at MNase-sensitive sites, which are more amenable to GGR activity, against the default association of unmodified XPC protein (devoid of ubiquitin) with MNase-resistant core particles. Accordingly, in the absence of CRL4^DDB2^ activity, more XPC protein binds to lesions located in nucleosome core particles (133). Later reports demonstrated that XPC protein displays an intrinsic avidity for histones (see below), suggesting that the apparent discrepancy between the findings obtained with the recombinant histones of reconstituted nucleosome core particles (to which XPC protein binds poorly) and the respective nucleoprotein complexes extracted from cells (to which XPC protein binds abundantly) may be explained by the existence of specific post-translational histone modifications occurring in living cells.

## AFFINITY OF XPC PROTEIN FOR HISTONES

**Figure 2.**
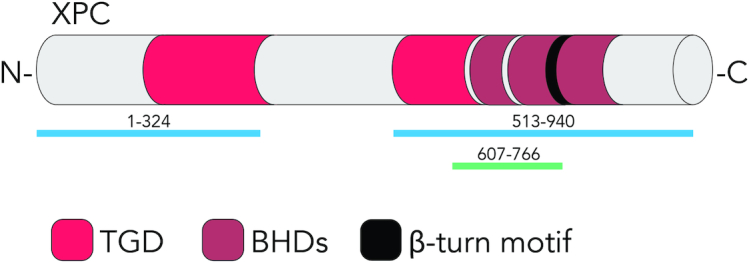
Interaction of XPC protein with histone H3. The domain structure of human XPC is shown with the proposed histone-interacting regions XPC_1–324_ and XPC_513–940_ (49) and the histone-interacting fragment XPC_607–766_ containing the β-turn motif (134). TGD, transglutaminase-like domain; BHD, β-hairpin domains. This initiator of GGR activity emerged as a ‘reader’ of histone methylation marks.

Immunoprecipitation studies confirmed that XPC protein coexists in nucleoprotein complexes together with histones H1, H3 and H4 (49,134). A direct interaction with histones H1 and H3, extracted from human fibroblasts or from recombinant sources, was demonstrated in biochemical assays based on far-Western blots (49). Interestingly, XPC-histone associations are dampened when this far-Western assay was performed using hyper-acetylated histones obtained from cells treated with the histone deacetylation inhibitor trichostatin A. Next, different truncated versions of human XPC protein were subjected to the far-Western assay, thus suggesting that amino acid residues at the N-terminus constitute a possible domain for contacts with H1 and H3 (Figure 2). In parallel, pull-down assays with histone H3 and truncated XPC protein indicated that another histone-interacting domain may reside in the C-terminus. Interestingly, the formation of XPC-histone complexes, determined in pull-down assays, was weakened by H3 acetylation at positions lysine 14 and lysine 27 (49).

Interactions between XPC and histones were also evidenced after the release of core particles by MNase digestion (134). This treatment generates soluble mono-nucleosome core particles amenable to immunoprecipitation using, for example, antibodies against histone H3. Analysis of the composition of immunoprecipitated complexes confirmed not only an increased appearance of DDB2 following UV radiation but also the presence of XPC protein. It remained possible that the interaction of XPC protein with core particles is mediated by its association with DNA. To rule out this, solubilized core particles were digested by benzonase, revealing that the absence of DNA around histone octamers does not reduce the amount of associated XPC protein. On the contrary, the benzonase treatment increased the interaction of XPC protein with the remaining DNA-free histone octamers.

To further test the hypothesis of a direct XPC–histone interaction, recombinant XPC protein from insect cell lysates was incubated with recombinant histones (the H3K4me3 form was used for these experiments instead of unmodified H3 due to the comparably higher efficiency of immunoprecipitation with the respective antibody) (134). This pull-down led to the co-isolation of XPC protein, thus lending further support to a direct interaction between XPC protein and histones. A β-turn motif (residues 741–757 of the human XPC protein) does not make contacts with DNA (71), but displays two negatively charged amino acids (positions 748 and 755) that may interact with positively charged histone residues (Figure 2). The prediction that the affinity for histones might be diminished by replacing negatively charged amino acids with positively charged counterparts was confirmed by expressing, in XP-C fibroblasts, mutant XPC containing charge inversions. By immunoprecipitation, it was shown that the D748K mutation reduces the ability of XPC protein to associate with histone H3. These same XPC constructs were used to complement the GGR defect of UV-irradiated XP-C fibroblasts, revealing that the reduced histone binding of the D748K mutant correlates with impaired CPD repair. To confirm that the β-turn (residues 741–757) participates in histone binding, it was shown that a polypeptide spanning XPC residues 607–766 (including the β-turn) but not the shorter polypeptide 607–741 (without the β-turn) retained the capacity for histone binding. Collectively, the studies of Kakumu *et al.* (49) and Balbo Pogliano *et al.* (134) suggest that XPC protein displays multiple interfaces for interactions with histones.

## GGR TRANSACTIONS ON A ‘CHROMATINIZED’ SUBSTRATE

The enzymatic GGR steps require that histones are temporary released from the DNA substrate by chromatin remodeling complexes, which use the energy of ATP hydrolysis to eject, slide, reorganize or replace nucleosomes (19,135–137). The DDB2 damage receptor, alone or in combination with the DDB1 adaptor, recruits at least two chromatin remodelers, i.e. ALC1 (Amplified in Liver Cancer 1) (138) and INO80 complex (INOsitol requiring 80) (139) to UV lesion sites. In isolation, these reports on the contribution of remodelers tend to suggest that nucleosomes need to be quickly displaced before recruitment of XPC protein, which serves as the initiator of GGR activity. However, there is also evidence indicating that chromatin remodelers are involved in downstream GGR reactions taking place after the association of XPC protein with damaged sites.

One example is provided by Rüthemann *et al.* (140), who found that the CHD1 (Chromodomain Helicase DNA-binding 1) remodeler stimulates a substrate handover from XPC to the TFIIH complex taking place explicitly on nucleosome core particles. In response to UV irradiation, CHD1 is recruited to nucleosome cores and depletion experiments with small interfering RNA (siRNA) demonstrated that this damage-dependent involvement of CHD1 relies on the prior recognition of lesion sites by XPC protein. The impact of CHD1 on the GGR pathway was analyzed by *in situ* immunofluorescence analyses of subnuclear UV lesion spots (generated by irradiation through micropore filters). This approach revealed that XPC protein accumulates at lesion sites even in the absence of CHD1, whereas the recruitment of TFIIH subunits is reduced, indicating that CHD1 promotes the transition between XPC bound to nucleosomes and the TFIIH complex (140). Whether CHD1 displaces the nucleosomes while facilitating this GGR transition is not known, but exactly this mechanism has been proposed for CHD1 during transcription initiation (141). In this early transcription stage, CHD1 associates with the most promoter-proximal nucleosome of active genes and evicts this nucleosome to allow for promoter escape by RNA Pol II. In the absence of CHD1, RNA Pol II remains sequestered on this promoter-proximal nucleosome. By analogy, in the absence of CHD1, XPC protein becomes sequestered on lesion sites on nucleosomes, without being able to give way to the follow-up step involving nucleosome eviction and TFIIH recruitment. Consequently, the depletion of CHD1 slows down CPD excision (but not the excision of 6–4PPs) and sensitizes cells to UV-induced cytotoxicity. The observation that a dynamic interaction between XPC, CHD1 and TFIIH takes place on nucleosome core particles strengthens the notion that chromatin is not simply an impediment to CPD recognition, but acts as a structural scaffold facilitating damage recognition and GGR transitions (140).

The second example of a chromatin remodeler involved in downstream GGR reactions is BRG1 (Brahma-Related Gene 1), which constitutes the catalytic ATPase subunit of the SWI/SNF (SWItching defective/Sucrose Non-Fermenting) complex, implicated in chromatin relaxation occurring after UV irradiation (142). The literature offers conflicting reports as to the precise involvement of SWI/SNF and its BRG1 subunit in the GGR pathway. Zhang *et al.* (143) found by immunofluorescence that BRG1 is recruited to spots of UV damage and that small hairpin RNA (shRNA)-mediated depletions of BRG1 reduce XPC accumulation in these damaged sites. An interaction between BRG1 and XPC was detected by Zhao *et al.* (142), but their siRNA-mediated depletion experiments indicated that the damage-dependent recruitment of BRG1 requires the prior recognition of lesion sites by XPC protein. This mode of SWI/SNF recruitment is consistent with the previously described interactions between SWI/SNF and the Rad4-Rad23 complex, which is the yeast homolog of human XPC-RAD23B (135). Next, Zhao *et al.* monitored the impact of a BRG1 depletion on the recruitment of up- and downstream GGR factors to UV lesion spots. They observed that, in the absence of BRG1, the accumulation of DDB2, XPC and XPB (a subunit of the TFIIH complex) to lesion sites is unaffected whereas the recruitment of the more downstream factors XPG and PCNA is reduced. As was observed in CHD1-depleted cells, the lack of BRG1 inhibits the excision of CPDs but not the excision of 6–4PPs (142,143). Based on these results, it can be concluded that BRG1 stimulates the repair of CPDs by supporting later stages of the GGR reaction, again suggesting that part of the GGR pathway in response to CPDs is executed on a nucleosome scaffold.

## PRIMING OF THE NUCLEOSOME SCAFFOLD BY HISTONE METHYLTRANSFERASES

A breakthrough in our understanding of GGR activity in chromatin came from the discovery of a new layer of regulation by lysine methyltransferases (KMTs), which use *S*-adenosylmethionine to add methyl groups to lysines of histones. Each of the core histones are folded in a globular core domain flanked by flexible tails, all presenting lysine residues that, by virtue of their positive charge, interact electrostatically with the negatively charged DNA backbone. These lysines are the target of posttranslational modifications including acetylation and methylation (144–148). By acetylation, the positive charge is masked, which weakens the electrostatic histone-DNA interactions, thus relaxing chromatin and exposing the double helix to DNA-binding proteins (50,149). Instead, lysine methylation does not change the histone charge but methylated side chains provide hydrophobic docking sites for downstream ‘reader’ proteins that induce condensation or relaxation of nucleosome arrays (146,149,150). The two modifications (acetylation and methylation) cannot occur simultaneously on the same lysine. Conversely, each lysine can accept up to three methyl groups, generating different degrees of methylation: mono- (me1), di- (me2) or trimethylated (me3). Distinctive histone modification patterns, for example di- or trimethylated H3K4, H3K36 and H3K79, or monomethylated H4K20, have been associated with the fluid chromatin of expressed genes. Instead, trimethylated H3K9, H3K27 and H4K20 correlate with the condensed chromatin of silenced genes. The first hint that histone methylation may regulate GGR activity came from the discovery in *D. melanogaster* that, after UV radiation, there is a decrease of H3K9me3 in the polytene chromosomes of salivary glands. This demethylation reaction is triggered by Dmp53 (the *D. melanogaster* p53 homolog), which induces expression of *KDM4B* (lysine demethylase 4B) that, in turn, demethylates H3K9me3. Larvae deficient in KDM4B activity display UV hypersensitivity and reduced CPD excision (151). This effect of a demethylase suggests that an adjustment of histone methylation may favor GGR activity in the chromatin context.

**Figure 3.**
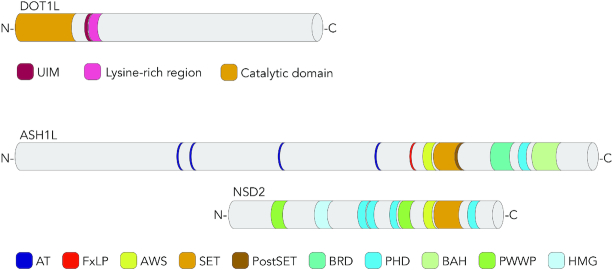
Overview of histone KMTs stimulating the GGR reaction in response to UV damage. DOT1L is the only known seven-β-strand methylase able to methylate lysine residues. Its catalytic domain, containing the seven-β-strand fold, is flanked by a ubiquitin-interacting motif (UIM) mediating its binding to ubiquitinated H2B (152,225,226). The lysine-rich region also participates in the interaction with ubiquitinated H2B and contributes to H3K79 methylation (227). ASH1L and NSD2 are members of a large family of SET-domain methyltransferases (146). The SET domain flanked by AWS (Associated With SET) forms the catalytic site. The post-SET motif displays a flexible auto-inhibitory loop (188–190). Further domains are the ‘AT hooks’ (putative DNA-binding motifs), BRD (a BRomoDomain interacting with acetylated histones), PHD (Plant HomeoDomain for the binding to methylated histones), BAH (a Bromo-Associated Homology domain for the binding to methylated histones and other proteins), PWWP (Pro-Trp-Trp-Pro domain for the binding to methylated histones) and an HMG box (for High Mobility Group, shown to interact with the DNA binding domain of the androgen receptor) (146,152,228,229). The FxLP motif in ASH1L mediates interactions with MRG15, a chromodomain protein stimulating ASH1L activity by releasing its auto-inhibitory loop.

KMTs responsible for the *de novo* deposition of methylation marks have been categorized into two evolutionary conserved groups depending on whether their catalytic center displays a seven-β-strand fold or a SET motif (Figure 3). The term ‘seven-β-strand’ describes a widespread methylase domain that consists of seven β-sheets (152). The SET motif has been named after the *Drosophila* genes *Su(var)3–9*, *Enhancer-of-zeste* and *Trithorax* (150,153,154) and the paradigm of SETD7, a prototypical KMT, demonstrates that the lysine methylation activity is not necessarily restricted to histones as this posttranslational modification regulates the stability and function of a much wider range of proteins (155). At least three lines of evidence support the conclusion that histone KMTs drive the recognition of UV lesions at distinct stages of the GGR pathway.

### Roles of DOT1L in the UV damage response

The seven-β-strand methylases DOT1 (Disruptor Of Telomeric silencing 1) in lower eukaryotes and DOT1L (DOT1-Like, also known as KMT4) in mammals are solely responsible for the mono-, di- and trimethylation of the lysine 79 residue of histone H3 (156,157). Of note, the ubiquitination of H2B at lysine 123 in yeast and lysine 120 in humans precedes H3K79 methylation and these modifications lie in close proximity within nucleosomes (158–161). It has been proposed that methylated H3K79, located in the histone globular domain, is not accessible under unchallenged conditions but becomes exposed when the chromatin structure is altered for example after DNA damage induction (162).

The *DOT1* gene was originally identified in a genetic screen in the yeast *Saccharomyces cerevisiae* because, when over-expressed, the DOT1 enzyme activates genes mapping to heterochromatic regions of telomeres (163). DOT1/DOT1L-mediated histone methylation has been associated with open chromatin during transcription (164–169) and, in many organisms, a defect in this histone methylation reaction reduces the survival after genotoxic attack, pinpointing to a function of DOT1/DOT1L in DNA damage responses (158,162,168,170,171). The methylation of H3K79 by DOT1 is also part of the UV radiation response in *S. cerevisiae* (172,173) and in the amoeba *Dictyostelium discoideum* (174). Based on the finding that DOT1-mediated methylation of H3K79 stimulates GGR activity in yeast, Tatum and Li (175) proposed that this particular histone mark may provide a docking site for the GGR complex in chromatin. *DOT1L^–/–^* mouse embryos grow slowly and die before completing their development (176). Additionally, DOT1L plays an important role in maintaining normal erythropoiesis in adults (156,177,178). The human *DOT1L* gene is frequently mutated or deleted in melanoma and many of the detected missense mutations display the UV signature, i.e. consist of C to T transitions at di-pyrimidine sites as well as tandem CC to TT transitions (179). However, the role of DOT1L in the mammalian UV radiation response remains unclear.

As observed in lower eukaryotes, the absence of DOT1L in mammalian cells confers UV hypersensitivity. Oksenych *et al.* (180) found in mouse embryonic fibroblasts that DOT1L supports transcription re-initiation following DNA damage excision, but without influencing GGR or TCR activity. These authors used fluorescence-based protein dynamics studies to show that DOT1L promotes the binding of RNA Pol II to chromatin during the recovery from UV irradiation. Concomitant ChIP studies indicated that, to re-initiate transcription after UV irradiation, DOT1L installs a transcription-permissive promoter structure characterized by H3K79me2 and acetylated histone H4, accompanied by a low level of the heterochromatin mark H3K9me2, which altogether facilitate the recruitment of transcription factors driving RNA Pol II. This role in the reactivation of transcription is reminiscent of the function of the histone chaperones FACT and HIRA, which are also dispensable for DNA repair but support transcription recovery after exposure to UV light (41,47).

Similarly, Zhu *et al.* (179) reported that an shRNA-mediated depletion of DOT1L in primary human melanocytes as well as loss-of-function *DOT1L* mutations in melanoma cells impart UV hypersensitivity. These authors monitored the excision of UV lesions and found that a deficiency of this enzyme (resulting from the shRNA-mediated depletion, its inactivation by loss-of-function mutations or a CRISPR–Cas9-engineered gene deletion) reduces the excision of both CPDs and 6–4PPs in melanocytes or melanoma cells. A caveat in these findings is that Zhu and colleagues observed an unusually fast excision with half-lives in the range of 2 h for CPDs and less than 1 h for 6–4PPs. With exceptions (181), other reports indicate that CPDs are removed from human cells with a longer half-life of ∼24 h (182). A possible explanation of the observed short half-lives is the relatively low UV dose yielding less CPDs and 6–4PPs and, of course, it is possible that melanocytes display an extraordinary UV lesion repair rate considering their physiologic role in protecting the skin from UV light. Nevertheless, it would have been helpful if Zhu *et al.* validated the excision assay with control cells lacking a core repair factor like for example XPA or XPC, to demonstrate that the observed loss of UV photoproducts is indeed due to NER activity. On the other hand, the same authors elaborated on the docking hypothesis formulated above and showed by immunofluorescence and chromatin fractionation that DOT1L promotes the recruitment of XPC protein and downstream factors, but not the recruitment of the upstream DDB2 subunit, to UV lesion sites. Intriguingly, there is no extra UV-dependent recruitment of DOT1L to chromatin in addition to its constitutive chromatin occupancy. Accordingly, UV irradiation does not increase the degree of H3K79 methylation. By immunoprecipitation, it was shown that XPC protein locates to chromatin complexes together with DOT1L and H3K79me2 (an enzymatic product of DOT1L). In line with a role of this histone methylation in the GGR function, conditional-null mice with a *DOT1L* deletion in melanocytes provide a very efficient cancer model with melanoma being induced in nearly 50% of animals by 10 weeks after UV irradiation (179). Although the participation of a constitutive H3K79 methylation in the UV damage response is clearly established, the impact of this histone mark is not fully understood but may reside in its capacity to attract downstream ‘reader’ proteins.

### ASH1L as a matchmaker during the DDB2 to XPC handoff at UV lesions

The histone methyltransferase ASH1 (Absent, Small or Homeotic discs 1) was identified in *D. melanogaster* as a member of the trithorax group that stimulates transcription by competing with the Polycomb silencing system and thereby regulates developmental *Hox* genes (183). An ASH1 deficiency causes homeotic transformations like antenna to leg, genitalia to leg or one thoracic segment to another (184–187). Mammalian ASH1L (ASH1-like, also known as KMT2H) is a SET-domain protein with histone H3 methyltransferase activity (Figure 3). The catalytic region comprises a core SET domain and a shell of flanking regulatory domains, including the post-SET domain forming an auto-inhibitory loop (188–190), but the exact lysine target of this enzyme is still debated. Several groups reported that ASH1L generates H3K36me2 (188,191–194), whereas others identified H3K4 as the methylation target (195,196). *ASH1L* is essential in mice (197) and hypomorphic mutants result in malformations or dysfunctions affecting the axial skeleton and the central nervous system (198,199). The ASH1L activity is also important for fertility (200), hematopoiesis and organ function in adults (196,201). Accordingly, ASH1L is expressed in many tissues (195,202). A role of ASH1L in the adult skin of mice is demonstrated by a mutation that causes keratinocyte hyperplasia, impaired epidermal stratification and defective wound healing (203). Overexpression of ASH1L is recurrent in breast cancer and cancer-associated *ASH1L* mutations have been identified in melanoma, breast, prostate, colon, liver and oesophageal cancer (204–207).

Balbo Pogliano *et al.* (134) identified ASH1L as an accessory GGR player driving the substrate handover from DDB2 to XPC protein. Biochemical fractionation revealed that, like DDB2 and XPC, ASH1L associates with chromatin following UV radiation. Depletion experiments showed that the DDB2 lesion receptor, independently of CRL4 ubiquitin ligase activity, is responsible for an additional recruitment of ASH1L to the chromatin of UV-irradiated cells over the constitutive presence of this methyltransferase in the chromatin of unchallenged cells. Immunoprecipitation of DDB2 from cell extracts or solubilized chromatin results in ASH1L co-precipitation, indicating that an interaction occurs between these two factors such that DDB2 mediates the relocation of ASH1L to UV lesion sites. The down regulation of ASH1L markedly reduces CPD excision in HeLa and U2OS cells but has no effect on the excision of 6-4PPs. This dampened CPD excision in ASH1L-depleted cells translates to lower repair patch synthesis measured after a 2-h recovery after UV irradiation, i.e. when most 6–4PPs have been removed. The role of ASH1L in GGR activity is confirmed by the observation that its down regulation confers UV hypersensitivity. Conversely, ASH1L is not involved in the TCR pathway because transcription recovery after UV radiation remains unaffected in ASH1L-depleted cells. In view of the finding that ASH1L stimulates GG-NER but not TC-NER activity, it was next demonstrated that this KMT does not negatively regulate the expression of XPC and DDB2, which are solely required for the GGR pathway (134). To understand how ASH1L regulates GGR activity, U2OS cells were UV-irradiated through filters with 3-μm pores to generate ‘small’ spots of UV lesions. After 2-h incubations to allow for repair of 6–4PPs, cells were irradiated through 5-μm pores to induce ‘big’ spots of UV lesions. Following another 15-min incubation, the cells were analyzed for XPC relocations. As expected, XPC protein in control cells is able to engage with not yet processed CPDs in small spots as well as with newly formed UV lesions, including 6–4PPs, in big spots. Upon ASH1L depletion, however, XPC is almost completely relocated to the big spots, indicating that in the absence of ASH1L the XPC protein is not able to stably interact with CPD sites and, as a consequence, readily moves to 6–4PPs in the newly formed big spots. This XPC protein dynamics suggests that, presumably by methylating histones, ASH1L is needed to generate a docking site for XPC protein in the proximity to CPD sites. This role in securing a stable XPC positioning was confirmed biochemically by monitoring the amount of this protein associated with the chromatin of UV irradiated cells. Additionally, in the absence of ASH1L, XPC protein is impaired in its ability to recruit the lesion verifier XPD, a subunit of the TFIIH complex. These findings are in line with the hypothesis that ASH1L is required to regulate the handoff between DDB2 and XPC, such that the latter is positioned in a robust association with CPD sites that allows for the TFIIH recruitment. It remains to be seen what is the critical methylation target in the GGR pathway and how ASH1L could facilitate the ‘hopping’ of XPC towards CPD lesions (see section on ‘Mammalian core NER reaction’ above) in the chromatin context.

### NSD2 as an auxiliary factor promoting the recruitment of XPA to damaged sites

The histone methyltransferase NSD2 (Nuclear receptor-binding SET Domain 2; also known as MMSET for Multiple Myeloma SET or WHSC1 for Wolf-Hirschhorn Syndrome Candidate 1) was first identified by its frequent rearrangement with the immunoglobulin locus in multiple myeloma patients (208,209) and, independently, by its role in the Wolf-Hirschhorn malformation syndrome (210,211). Amplifications, gain-of-function mutations or translocations of *NSD2* are linked to numerous types of cancer (212,213). Mice with homozygous deletions of this gene do not survive longer than 10 days after birth. Indeed, the NSD2 methyltransferase regulates crucial developmental genes including *Sall1*, *Sall4* or *Nanog* (214). NSD2 is additionally involved in the repair of DSBs and antibody class switching recombination (215,216). Moreover, NSD2 interacts with HIRA and like this chaperone is required for the deposition of the H3.3 histone variant (217).

Elaborating on their previous finding that the endoribonuclease Dicer associates with chromatin and thereby stimulates DNA repair patch synthesis in UV-irradiated cells, Chitale and Richly (218,219) reported a mechanism by which Dicer, independently of its ribonuclease activity, recruits the histone methyltransferase NSD2 to UV lesion sites. By immunofluorescence, these authors observed that NSD2 relocates to intranuclear spots of UV damage where it generates the histone mark H4K20me2. Although shRNA experiments indicate that NSD2 activity at UV lesion sites depends on DDB2 but is not affected by the depletion of XPC protein, an indispensable GGR core factor, the authors concluded that NSD2 acts by promoting GGR activity. In support of this view, Chitale and Richly reported that the knockdown of NSD2 reduces cell survival after UV irradiation and also diminishes the synthesis of DNA repair patches in UV lesion spots. Following NSD2 depletion, the excision of CPDs was indistinguishable from that in control cells during the first 24 h after UV irradiation, but then the rate of CPD excision was lower in NSD2-depleted cells than in controls between 24 and 48 h after UV irradiation. Next, the impact on various GGR factors was tested and the authors found that NSD2 stimulates the recruitment of XPA protein to intranuclear UV lesion spots. They describe a scenario in which DDB2 mediates the translocation of NSD2 to UV lesions by a multiprotein mechanism that starts with H2A ubiquitination and recruitment of the ubiquitin-binding protein ZRF1 (Zuotin-Related Factor 1), which interacts with Dicer. In turn, Dicer provides an interaction partner for NSD2, resulting in histone H4 methylation to generate H4K20me2. The authors further argue that di-methylation of histone H4 generates a docking point for 53BP1, which is also a ‘reader’ of the H4K20me2 mark and, through interactions with RPA, this multiprotein network promotes the recruitment of XPA protein to lesion sites (219). The relevance of this protein network for the GGR process is not yet conclusively demonstrated, particularly in view of the finding that the assembly of this chromatin-associated complex, and the consequent recruitment of XPA, is triggered by DDB2 without participation of XPC protein, which is the indispensable initiator of GGR activity. Such a recruitment of XPA in the absence of XPC is generally not observed (66). Another caveat is that the purported contribution of NSD2 in H4K20 methylation, to promote 53BP1 recruitment to sites of DNA damage (215), remained controversial (220). Other KMTs have since then been implicated in this reaction (221,222). On the other hand, NSD2 is also capable of methylating H3K36 (223) and may therefore act in concert with DOT1L and ASH1L to modify histone H3 in response to UV radiation. In any case, the potential contribution of NSD2 in modulating the histone code at UV lesions confirms once more that the DNA substrate remains at least partially ‘chromatinized’ and reinforces the hypothesis that KMTs are needed to carry out the GGR process in the nucleosome landscape of chromatin.

## CONCLUSIONS

The GGR machine constitutes a major caretaker of the human genome, safeguarding cellular homeostasis against endogenous and exogenous genotoxic insults. For example, NER mitigates the negative effects of the UV radiation of sunlight including sunburns, skin photoaging and cutaneous cancer. The same DNA repair process is responsible for the excision of bulky DNA adducts elicited by tobacco smoke, food carcinogens and crosslinking drugs. The GGR pathway is also the sole DNA repair system available for the excision of bulky lesions resulting from endogenous metabolic byproducts like, in particular, cyclopurine adducts generated by reactive oxygen species (25). In view of the pathophysiologic consequences of persistently accumulating DNA damage, this excision repair response is essential to prevent chronic diseases like cancer and to protect from neurodegeneration and other debilitating traits of aging, which now belong to the most significant public health problems in Western societies. It is, therefore, important to understand the emerging role of KMTs like DOT1L, ASH1L and NSD2 in regulating the efficiency by which the GGR machine removes bulky DNA adducts. An outstanding question is to determine whether the relevant histone modifications are constitutive (as currently suggested for DOT1L) or induced around the lesions (as suggested for ASH1L and NSD2). It remains to be determined what are the critical methylation targets of these KMTs during the GGR process and how their interplay stimulates DNA damage recognition and downstream GGR reactions. Besides, the chromatin context should be taken into account and more particularly the presence of histones variants. For example, histone variant H3.3 is more easily found modified by DOT1L than the canonical histone H3.1 (224), which may give rise to combinatorial, possibly synergistic effects of histone variants and histone methylation in modulating GGR efficiency. Knowledge of the molecular mechanism by which KMTs fine-tune GGR activity, and thereby mitigate DNA damage accumulation, cell cycle checkpoints, apoptosis and mutagenesis, will pave the way to the identification of new drug targets and the development of novel therapeutic approaches for the management of cancer and other chronic conditions. We would envision, for example, a strategy where a specific KMT deficiency might be counteracted or at least mitigated through the maintenance of minimal methylation levels by deactivating appropriate lysine demethylases with selective inhibitors.

## References

[1] Hoeijmakers J.H.J. DNA damage, aging, and cancer. N. Engl. J. Med. 2009; 361:1475–1485. 19812404 10.1056/NEJMra0804615

[2] Diderich K., Alanazi M., Hoeijmakers J.H.J. Premature aging and cancer in nucleotide excision repair-disorders. DNA Repair (Amst). 2011; 10:772–780. 21680258 10.1016/j.dnarep.2011.04.025 PMC4128095

[3] Schärer O.D. Nucleotide excision repair in eukaryotes. Cold Spring Harb. Perspect. Biol. 2013; 5:a012609. 24086042 10.1101/cshperspect.a012609 PMC3783044

[4] Mouret S., Baudouin C., Charveron M., Favier A., Cadet J., Douki T. Cyclobutane pyrimidine dimers are predominant DNA lesions in whole human skin exposed to UVA radiation. Proc. Natl. Acad. Sci. U.S.A. 2006; 103:13765–13770. 16954188 10.1073/pnas.0604213103 PMC1564232

[5] Mouret S., Bogdanowicz P., Haure M.-J., Castex-Rizzi N., Cadet J., Favier A., Douki T. Assessment of the photoprotection properties of sunscreens by chromatographic measurement of DNA damage in skin explants. Photochem. Photobiol. 2011; 87:109–116. 21091484 10.1111/j.1751-1097.2010.00834.x

[6] Cadet J., Douki T. Formation of UV-induced DNA damage contributing to skin cancer development. Photochem. Photobiol. Sci. 2018; 17:1816–1841. 29405222 10.1039/c7pp00395a

[7] McGinty R.K., Tan S. Nucleosome structure and function. Chem. Rev. 2015; 115:2255–2273. 25495456 10.1021/cr500373h PMC4378457

[8] Zhou K., Gaullier G., Luger K. Nucleosome structure and dynamics are coming of age. Nat. Struct. Mol. Biol. 2019; 26:3–13. 30532059 10.1038/s41594-018-0166-x PMC7386248

[9] Armeev G.A., Gribkova A.K., Pospelova I., Komarova G.A., Shaytan A.K. Linking chromatin composition and structural dynamics at the nucleosome level. Curr. Opin. Struct. Biol. 2019; 56:46–55. 30529788 10.1016/j.sbi.2018.11.006

[10] Mao P., Brown A.J., Esaki S., Lockwood S., Poon G.M.K., Smerdon M.J., Roberts S.A., Wyrick J.J. ETS transcription factors induce a unique UV damage signature that drives recurrent mutagenesis in melanoma. Nat. Commun. 2018; 9:2626. 29980679 10.1038/s41467-018-05064-0 PMC6035183

[11] Brown A.J., Mao P., Smerdon M.J., Wyrick J.J., Roberts S.A. Nucleosome positions establish an extended mutation signature in melanoma. PLoS Genet. 2018; 14:e1007823. 30485262 10.1371/journal.pgen.1007823 PMC6287878

[12] Adar S., Hu J., Lieb J.D., Sancar A. Genome-wide kinetics of DNA excision repair in relation to chromatin state and mutagenesis. Proc. Natl. Acad. Sci. U.S.A. 2016; 113:E2124–E2133. 27036006 10.1073/pnas.1603388113 PMC4839430

[13] Li W., Hu J., Adebali O., Adar S., Yang Y., Chiou Y.-Y., Sancar A. Human genome-wide repair map of DNA damage caused by the cigarette smoke carcinogen benzo[a]pyrene. Proc. Natl. Acad. Sci. U.S.A. 2017; 114:6752–6757. 28607059 10.1073/pnas.1706021114 PMC5495276

[14] Hu J., Adebali O., Adar S., Sancar A. Dynamic maps of UV damage formation and repair for the human genome. Proc. Natl. Acad. Sci. U.S.A. 2017; 114:6758–6763. 28607063 10.1073/pnas.1706522114 PMC5495279

[15] Smerdon M.J., Conconi A. Modulation of DNA damage and DNA repair in chromatin. Prog. Nucleic Acid Res. Mol. Biol. 1998; 62:227–255. 10.1016/s0079-6603(08)60509-7 9932456

[16] Mao P., Smerdon M.J., Roberts S.A., Wyrick J.J. Chromosomal landscape of UV damage formation and repair at single-nucleotide resolution. Proc. Natl. Acad. Sci. U.S.A. 2016; 113:9057–9062. 27457959 10.1073/pnas.1606667113 PMC4987812

[17] Wang Z., Wu X., Friedberg E.C. Nucleotide excision repair of DNA by human cell extracts is suppressed in reconstituted nucleosomes. J. Biol. Chem. 1991; 266:22472–22478. 1939267

[18] Hara R., Mo J., Sancar A. DNA damage in the nucleosome core is refractory to repair by human excision nuclease. Mol. Cell. Biol. 2000; 20:9173–9181. 11094069 10.1128/mcb.20.24.9173-9181.2000 PMC102175

[19] Ura K., Araki M., Saeki H., Masutani C., Ito T., Iwai S., Mizukoshi T., Kaneda Y., Hanaoka F. ATP-dependent chromatin remodeling facilitates nucleotide excision repair of UV-induced DNA lesions in synthetic dinucleosomes. EMBO J. 2001; 20:2004–2014. 11296233 10.1093/emboj/20.8.2004 PMC125421

[20] Liu X., Smerdon M.J. Nucleotide excision repair of the 5 S ribosomal RNA gene assembled into a nucleosome. J. Biol. Chem. 2000; 275:23729–23735. 10821833 10.1074/jbc.M002206200

[21] Nag R., Smerdon M.J. Altering the chromatin landscape for nucleotide excision repair. Mutat. Res. Mutat. Res. 2009; 682:13–20. 19167517 10.1016/j.mrrev.2009.01.002

[22] Adam S., Dabin J., Chevallier O., Leroy O., Baldeyron C., Corpet A., Lomonte P., Renaud O., Almouzni G., Polo S.E. Real-time tracking of parental histones reveals their contribution to chromatin integrity following DNA damage. Mol. Cell. 2016; 64:65–78. 27642047 10.1016/j.molcel.2016.08.019 PMC5065526

[23] Mu D., Park C.-H., Matsunaga T., Hsu D.S., Reardon J.T., Sancar A. Reconstitution of human DNA repair excision nuclease in a highly defined system. J. Biol. Chem. 1995; 270:2415–2418. 7852297 10.1074/jbc.270.6.2415

[24] Aboussekhra A., Biggerstaff M., Shivji M.K.K., Vilpo J.A., Moncollin V., Podust V.N., Protić M., Hübscher U., Egly J.-M., Wood R.D. Mammalian DNA nucleotide excision repair reconstituted with purified protein components. Cell. 1995; 80:859–868. 7697716 10.1016/0092-8674(95)90289-9

[25] Marteijn J.A., Lans H., Vermeulen W., Hoeijmakers J.H.J. Understanding nucleotide excision repair and its roles in cancer and ageing. Nat. Rev. Mol. Cell Biol. 2014; 15:465–481. 24954209 10.1038/nrm3822

[26] Czaja W., Mao P., Smerdon M.J., Czaja W., Mao P., Smerdon M.J. The emerging roles of ATP-dependent chromatin remodeling enzymes in nucleotide excision repair. Int. J. Mol. Sci. 2012; 13:11954–11973. 23109894 10.3390/ijms130911954 PMC3472786

[27] Palomera-Sanchez Z., Zurita M. Open, repair and close again: chromatin dynamics and the response to UV-induced DNA damage. DNA Repair (Amst). 2011; 10:119–125. 21130713 10.1016/j.dnarep.2010.10.010

[28] Polo S.E., Almouzni G. Chromatin dynamics after DNA damage: the legacy of the access–repair–restore model. DNA Repair (Amst). 2015; 36:114–121. 26429064 10.1016/j.dnarep.2015.09.014 PMC5113751

[29] Telford D.J., Stewart B.W. Characteristics of chromatin release during digestion of nuclei with micrococcal nuclease: preferential solubilization of nascent RNA at low enzyme concentration. Int. J. Biochem. 1989; 21:1235–1240. 2482203 10.1016/0020-711x(89)90009-8

[30] Smerdon M.J., Lieberman M.W. Nucleosome rearrangement in human chromatin during UV-induced DNA-repair synthesis. Proc. Natl. Acad. Sci. U.S.A. 1978; 75:4238–4141. 279912 10.1073/pnas.75.9.4238 PMC336087

[31] Zavala A.G., Morris R.T., Wyrick J.J., Smerdon M.J. High-resolution characterization of CPD hotspot formation in human fibroblasts. Nucleic Acids Res. 2014; 42:893–905. 24137003 10.1093/nar/gkt912 PMC3902913

[32] Matsumoto S., Cavadini S., Bunker R.D., Grand R.S., Potenza A., Rabl J., Yamamoto J., Schenk A.D., Schübeler D., Iwai S. et al. DNA damage detection in nucleosomes involves DNA register shifting. Nature. 2019; 571:79–84. 31142837 10.1038/s41586-019-1259-3 PMC6611726

[33] Gaillard P.-H.L., Martini E.M.-D., Kaufman P.D., Stillman B., Moustacchi E., Almouzni G. Chromatin assembly coupled to DNA repair: a new role for chromatin assembly factor I. Cell. 1996; 86:887–896. 8808624 10.1016/s0092-8674(00)80164-6

[34] Gaillard P.-H.L., Moggs J.G., Roche D.M.J., Quivy J., Becker P.B., Wood R.D., Almouzni G. Initiation and bidirectional propagation of chromatin assembly from a target site for nucleotide excision repair. EMBO J. 1997; 16:6281–6289. 9321407 10.1093/emboj/16.20.6281 PMC1326312

[35] Martini E., Roche D.M.J., Marheineke K., Verreault A., Almouzni G. Recruitment of phosphorylated chromatin assembly factor 1 to chromatin after UV irradiation of human cells. J. Cell Biol. 1998; 143:563–575. 9813080 10.1083/jcb.143.3.563 PMC2148138

[36] Moggs J.G., Grandi P., Quivy J.-P., Jonsson Z.O., Hübscher U., Becker P.B., Almouzni G. A CAF-1-PCNA-mediated chromatin assembly pathway triggered by sensing DNA damage. Mol. Cell. Biol. 2000; 20:1206–1218. 10648606 10.1128/mcb.20.4.1206-1218.2000 PMC85246

[37] Green C.M., Almouzni G. Local action of the chromatin assembly factor CAF-1 at sites of nucleotide excision repair in vivo. EMBO J. 2003; 22:5163–5174. 14517254 10.1093/emboj/cdg478 PMC204462

[38] Mello J.A., Silljé H.H.W., Roche D.M.J., Kirschner D.B., Nigg E.A., Almouzni G. Human Asf1 and CAF-1 interact and synergize in a repair-coupled nucleosome assembly pathway. EMBO Rep. 2002; 3:329–334. 11897662 10.1093/embo-reports/kvf068 PMC1084056

[39] Groth A., Rocha W., Verreault A., Ve Almouzni G., Almouzni G. Chromatin challenges during DNA replication and repair. Cell. 2007; 128:721–733. 17320509 10.1016/j.cell.2007.01.030

[40] Polo S.E., Roche D., Almouzni G. New histone incorporation marks sites of UV repair in human cells. Cell. 2006; 127:481–493. 17081972 10.1016/j.cell.2006.08.049

[41] Dinant C., Ampatziadis-Michailidis G., Lans H., Tresini M., Lagarou A., Grosbart M., Theil A.F., van Cappellen W.A., Kimura H., Bartek J. et al. Enhanced chromatin dynamics by FACT promotes transcriptional restart after UV-induced DNA damage. Mol. Cell. 2013; 51:469–479. 23973375 10.1016/j.molcel.2013.08.007

[42] Piquet S., Le Parc F., Bai S.-K., Chevallier O., Adam S., Polo S.E. The histone chaperone FACT coordinates H2A.X-dependent signaling and repair of DNA damage. Mol. Cell. 2018; 72:888–901. 30344095 10.1016/j.molcel.2018.09.010 PMC6292839

[43] Gale J.M., Nissen K.A., Smerdon M.J. UV-induced formation of pyrimidine dimers in nucleosome core DNA is strongly modulated with a period of 10.3 bases. Proc. Natl. Acad. Sci. U.S.A. 1987; 84:6644–6648. 3477794 10.1073/pnas.84.19.6644 PMC299139

[44] Brown D.W., Libertini L.J., Suquet C., Small E.W., Smerdon M.J. Unfolding of nucleosome cores dramatically changes the distribution of ultraviolet photoproducts in DNA. Biochemistry. 1993; 32:10527–10531. 8399198 10.1021/bi00091a001

[45] Luijsterburg M.S., Dinant C., Lans H., Stap J., Wiernasz E., Lagerwerf S., Warmerdam D.O., Lindh M., Brink M.C., Dobrucki J.W. et al. Heterochromatin protein 1 is recruited to various types of DNA damage. J. Cell Biol. 2009; 185:577–586. 19451271 10.1083/jcb.200810035 PMC2711568

[46] Cheutin T., McNairn A.J., Jenuwein T., Gilbert D.M., Singh P.B., Misteli T. Maintenance of stable heterochromatin domains by dynamic HP1 binding. Science. 2003; 299:721–725. 12560555 10.1126/science.1078572

[47] Adam S., Polo S.E., Almouzni G. Transcription recovery after DNA damage requires chromatin priming by the H3.3 histone chaperone HIRA. Cell. 2013; 155:94–106. 24074863 10.1016/j.cell.2013.08.029

[48] Yang X., Li L., Liang J., Shi L., Yang J., Yi X., Zhang D., Han X., Yu N., Shang Y. Histone acetyltransferase 1 promotes homologous recombination in DNA repair by facilitating histone turnover. J. Biol. Chem. 2013; 288:18271–18282. 23653357 10.1074/jbc.M113.473199 PMC3689969

[49] Kakumu E., Nakanishi S., Shiratori H.M., Kato A., Kobayashi W., Machida S., Yasuda T., Adachi N., Saito N., Ikura T. et al. Xeroderma pigmentosum group C protein interacts with histones: Regulation by acetylated states of histone H3. Genes Cells. 2017; 22:310–327. 28233440 10.1111/gtc.12479

[50] Barnes C.E., English D.M., Cowley S.M. Acetylation & CO: an expanding repertoire of histone acylations regulates chromatin and transcription. Essays Biochem. 2019; 63:97–107. 30940741 10.1042/EBC20180061 PMC6484784

[51] Li Z., Mon H., Mitsunobu H., Zhu L., Xu J., Lee J.M., Kusakabe T. Dynamics of polycomb proteins-mediated histone modifications during UV irradiation-induced DNA damage. Insect Biochem. Mol. Biol. 2014; 55:9–18. 25308962 10.1016/j.ibmb.2014.10.001

[52] Chou D.M., Adamson B., Dephoure N.E., Tan X., Nottke A.C., Hurov K.E., Gygi S.P., Colaiácovo M.P., Elledge S.J. A chromatin localization screen reveals poly (ADP ribose)-regulated recruitment of the repressive polycomb and NuRD complexes to sites of DNA damage. Proc. Natl. Acad. Sci. U.S.A. 2010; 107:18475–18480. 20937877 10.1073/pnas.1012946107 PMC2972950

[53] Ui A., Nagaura Y., Yasui A. Transcriptional elongation factor ENL phosphorylated by ATM recruits polycomb and switches off transcription for DSB repair. Mol. Cell. 2015; 58:468–482. 25921070 10.1016/j.molcel.2015.03.023

[54] Mann D.B., Springer D.L., Smerdon M.J. DNA damage can alter the stability of nucleosomes: Effects are dependent on damage type. Proc. Natl. Acad. Sci. U.S.A. 1997; 94:2215–2220. 9122174 10.1073/pnas.94.6.2215 PMC20067

[55] Duan M.-R., Smerdon M.J. UV damage in DNA promotes nucleosome unwrapping. J. Biol. Chem. 2010; 285:26295–26303. 20562439 10.1074/jbc.M110.140087 PMC2924050

[56] Huang J.-C., Svoboda D.L., Reardon J.T., Sancar A. Human nucleotide excision nuclease removes thymine dimers from DNA by incising the 22nd phosphodiester bond 5′ and the 6th phosphodiester bond 3′ to the photodimer. Proc. Natl. Acad. Sci. U.S.A. 1992; 89:3664–3668. 1314396 10.1073/pnas.89.8.3664 PMC48929

[57] Moggs J.G., Yarema K.J., Essigmann J.M., Wood R.D. Analysis of incision sites produced by human cell extracts and purified proteins during nucleotide excision repair of a 1,3-intrastrand d(GpTpG)-cisplatin adduct. J. Biol. Chem. 1996; 271:7177–7186. 8636155 10.1074/jbc.271.12.7177

[58] Spivak G. Transcription-coupled repair: an update. Arch. Toxicol. 2016; 90:2583–2594. 27549370 10.1007/s00204-016-1820-x PMC5065778

[59] Portman J.R., Strick T.R. Transcription-coupled repair and complex biology. J. Mol. Biol. 2018; 430:4496–4512. 29733857 10.1016/j.jmb.2018.04.033

[60] Gregersen L.H., Svejstrup J.Q. The cellular response to transcription-blocking DNA damage. Trends Biochem. Sci. 2018; 43:327–341. 29699641 10.1016/j.tibs.2018.02.010 PMC5929563

[61] Mu H., Geacintov N.E., Broyde S., Yeo J.-E., Schärer O.D. Molecular basis for damage recognition and verification by XPC-RAD23B and TFIIH in nucleotide excision repair. DNA Repair (Amst). 2018; 71:33–42. 30174301 10.1016/j.dnarep.2018.08.005 PMC6340764

[62] DiGiovanna J.J., Kraemer K.H. Shining a light on xeroderma pigmentosum. J. Invest. Dermatol. 2012; 132:785–796. 22217736 10.1038/jid.2011.426 PMC3279615

[63] Lehmann J., Seebode C., Martens M.C., Emmert S. Xeroderma pigmentosum - facts and perspectives. Anticancer Res. 2018; 38:1159–1164. 29374753 10.21873/anticanres.12335

[64] Nishigori C., Nakano E., Masaki T., Ono R., Takeuchi S., Tsujimoto M., Ueda T. Characteristics of xeroderma pigmentosum in Japan: lessons from two clinical surveys and measures for patient care. Photochem. Photobiol. 2019; 95:140–153. 30565713 10.1111/php.13052

[65] Sugasawa K., Ng J.M.Y., Masutani C., Iwai S., van der Spek P.J., Eker A.P.M., Hanaoka F., Bootsma D., Hoeijmakers J.H.J. Xeroderma pigmentosum group C protein complex is the initiator of global genome nucleotide excision repair. Mol. Cell. 1998; 2:223–232. 9734359 10.1016/s1097-2765(00)80132-x

[66] Volker M., Moné M.J., Karmakar P., van Hoffen A., Schul W., Vermeulen W., Hoeijmakers J.H.J., van Driel R., van Zeeland A.A.A., Mullenders L.H.F. Sequential assembly of the nucleotide excision repair factors in vivo. Mol. Cell. 2001; 8:213–224. 11511374 10.1016/s1097-2765(01)00281-7

[67] Ng J.M.Y., Vermeulen W., van der Horst G.T.J., Bergink S., Sugasawa K., Vrieling H., Hoeijmakers J.H.J. A novel regulation mechanism of DNA repair by damage-induced and RAD23-dependent stabilization of xeroderma pigmentosum group C protein. Genes Dev. 2003; 17:1630–1645. 12815074 10.1101/gad.260003 PMC196135

[68] Nishi R., Okuda Y., Watanabe E., Mori T., Iwai S., Masutani C., Sugasawa K., Hanaoka F. Centrin 2 stimulates nucleotide excision repair by interacting with xeroderma pigmentosum group C protein. Mol. Cell Biol. 2005; 25:5664–5674. 15964821 10.1128/MCB.25.13.5664-5674.2005 PMC1156980

[69] Buterin T., Meyer C., Giese B., Naegeli H. DNA quality control by conformational readout on the undamaged strand of the double helix. Chem. Biol. 2005; 12:913–922. 16125103 10.1016/j.chembiol.2005.06.011

[70] Maillard O., Solyom S., Naegeli H. An aromatic sensor with aversion to damaged strands confers versatility to DNA repair. PLoS Biol. 2007; 5:e79. 17355181 10.1371/journal.pbio.0050079 PMC1820611

[71] Min J.-H., Pavletich N.P. Recognition of DNA damage by the Rad4 nucleotide excision repair protein. Nature. 2007; 449:570–575. 17882165 10.1038/nature06155

[72] Mu H., Geacintov N.E., Min J.-H., Zhang Y., Broyde S. Nucleotide excision repair lesion-recognition protein Rad4 captures a pre-flipped partner base in a benzo[a]pyrene-derived DNA lesion: how structure impacts the binding pathway. Chem. Res. Toxicol. 2017; 30:1344–1354. 28460163 10.1021/acs.chemrestox.7b00074 PMC5478902

[73] Araújo S.J., Tirode F., Coin F., Pospiech H., Syväoja J.E., Stucki M., Hübscher U., Egly J.M., Wood R.D. Nucleotide excision repair of DNA with recombinant human proteins: definition of the minimal set of factors, active forms of TFIIH, and modulation by CAK. Genes Dev. 2000; 14:349–359. 10673506 PMC316364

[74] van der Spek P.J., Eker A., Rademakers S., Visser C., Sugasawa K., Masutani C., Hanaoka F., Bootsma D., Hoeijmakers J.H.J. XPC and human homologs of RAD23: intracellular localization and relationship to other nucleotide excision repair complexes. Nucleic Acids Res. 1996; 24:2551–2559. 8692695 10.1093/nar/24.13.2551 PMC145966

[75] Camenisch U., Träutlein D., Clement F.C., Fei J., Leitenstorfer A., Ferrando-May E., Naegeli H. Two-stage dynamic DNA quality check by xeroderma pigmentosum group C protein. EMBO J. 2009; 28:2387–2399. 19609301 10.1038/emboj.2009.187 PMC2735174

[76] Cheon N.Y., Kim H.-S., Yeo J.-E., Schärer O.D., Lee J.Y. Single-molecule visualization reveals the damage search mechanism for the human NER protein XPC-RAD23B. Nucleic Acids Res. 2019; 47:8337–8347. 31372632 10.1093/nar/gkz629 PMC6895271

[77] Sugasawa K., Akagi J., Nishi R., Iwai S., Hanaoka F. Two-step recognition of DNA damage for mammalian nucleotide excision repair: Directional binding of the XPC complex and DNA strand scanning. Mol. Cell. 2009; 36:642–653. 19941824 10.1016/j.molcel.2009.09.035

[78] Dantas T.J., Wang Y., Lalor P., Dockery P., Morrison C.G. Defective nucleotide excision repair with normal centrosome structures and functions in the absence of all vertebrate centrins. J. Cell Biol. 2011; 193:307–318. 21482720 10.1083/jcb.201012093 PMC3080269

[79] Bergink S., Toussaint W., Luijsterburg M.S., Dinant C., Alekseev S., Hoeijmakers J.H.J., Dantuma N.P., Houtsmuller A.B., Vermeulen W. Recognition of DNA damage by XPC coincides with disruption of the XPC-RAD23 complex. J. Cell Biol. 2012; 196:681–688. 22431748 10.1083/jcb.201107050 PMC3308700

[80] Yokoi M., Masutani C., Maekawa T., Sugasawa K., Ohkuma Y., Hanaoka F. The xeroderma pigmentosum group C protein complex XPC-HR23B plays an important role in the recruitment of transcription factor IIH to damaged DNA. J. Biol. Chem. 2000; 275:9870–9875. 10734143 10.1074/jbc.275.13.9870

[81] Bernardes de Jesus B.M., Bjørås M., Coin F., Egly J.M. Dissection of the molecular defects caused by pathogenic mutations in the DNA repair factor XPC. Mol. Cell Biol. 2008; 28:7225–7235. 18809580 10.1128/MCB.00781-08 PMC2593387

[82] Oksenych V., de Jesus B.B., Zhovmer A., Egly J.-M., Coin F. Molecular insights into the recruitment of TFIIH to sites of DNA damage. EMBO J. 2009; 28:2971–2980. 19713942 10.1038/emboj.2009.230 PMC2760107

[83] Mathieu N., Kaczmarek N., Naegeli H. Strand- and site-specific DNA lesion demarcation by the xeroderma pigmentosum group D helicase. Proc. Natl. Acad. Sci. U.S.A. 2010; 107:17545–17550. 20876134 10.1073/pnas.1004339107 PMC2955138

[84] Mathieu N., Kaczmarek N., Rüthemann P., Luch A., Naegeli H. DNA quality control by a lesion sensor pocket of the xeroderma pigmentosum group D helicase subunit of TFIIH. Curr. Biol. 2013; 23:204–212. 23352696 10.1016/j.cub.2012.12.032

[85] Coin F., Oksenych V., Mocquet V., Groh S., Blattner C., Egly J.M. Nucleotide excision repair driven by the dissociation of CAK from TFIIH. Mol. Cell. 2008; 31:9–20. 18614043 10.1016/j.molcel.2008.04.024

[86] Kokic G., Chernev A., Tegunov D., Dienemann C., Urlaub H., Cramer P. Structural basis of TFIIH activation for nucleotide excision repair. Nat. Commun. 2019; 10:2885. 31253769 10.1038/s41467-019-10745-5 PMC6599211

[87] Evans E., Fellows J., Coffer A., Wood R.D. Open complex formation around a lesion during nucleotide excision repair provides a structure for cleavage by human XPG protein. EMBO J. 1997; 16:625–638. 9034344 10.1093/emboj/16.3.625 PMC1169665

[88] Wakasugi M., Sancar A. Assembly, subunit composition, and footprint of human DNA repair excision nuclease. Proc. Natl. Acad. Sci. U.S.A. 1998; 95:6669–6674. 9618470 10.1073/pnas.95.12.6669 PMC22593

[89] Missura M., Buterin T., Hindges R., Hübscher U., Kaspárková J., Brabec V., Naegeli H. Double-check probing of DNA bending and unwinding by XPA-RPA: An architectural function in DNA repair. EMBO J. 2001; 20:3554–3564. 11432842 10.1093/emboj/20.13.3554 PMC125508

[90] Li C.-L., Golebiowski F.M., Onishi Y., Samara N.L., Sugasawa K., Yang W. Tripartite DNA lesion recognition and verification by XPC, TFIIH, and XPA in nucleotide excision repair. Mol. Cell. 2015; 59:1025–1034. 26384665 10.1016/j.molcel.2015.08.012 PMC4617536

[91] Staresincic L., Fagbemi A.F., Enzlin J.H., Gourdin A.M., Wijgers N., Dunand-Sauthier I., Giglia-Mari G., Clarkson S.G., Vermeulen W., Schärer O.D. Coordination of dual incision and repair synthesis in human nucleotide excision repair. EMBO J. 2009; 28:1111–1120. 19279666 10.1038/emboj.2009.49 PMC2683701

[92] Ogi T., Limsirichaikul S., Overmeer R.M., Volker M., Takenaka K., Cloney R., Nakazawa Y., Niimi A., Miki Y., Jaspers N.G. et al. Three DNA polymerases, recruited by different mechanisms, carry out NER repair synthesis in human cells. Mol. Cell. 2010; 37:714–727. 20227374 10.1016/j.molcel.2010.02.009

[93] Moser J., Kool H., Giakzidis I., Caldecott K., Mullenders L.H.F., Fousteri M.I. Sealing of chromosomal DNA nicks during nucleotide excision repair requires XRCC1 and DNA ligase IIIα in a cell-cycle-specific manner. Mol. Cell. 2007; 27:311–323. 17643379 10.1016/j.molcel.2007.06.014

[94] Kim J.-K., Choi B.-S. The solution structure of DNA duplex-decamer containing the (6-4) photoproduct of thymidylyl(3′5′)thymidine by NMR and relaxation matrix refinement. Eur. J. Biochem. 1995; 228:849–854. 7737185 10.1111/j.1432-1033.1995.tb20331.x

[95] Jing Y., Taylor J.-S., Kao J.F.-L. Thermodynamic and base-pairing studies of matched and mismatched DNA dodecamer duplexes containing cis-syn, (6-4) and Dewar photoproducts of TT. Nucleic Acids Res. 1998; 26:3845–3853. 9685504 10.1093/nar/26.16.3845 PMC147757

[96] McAteer K., Jing Y., Kao J., Taylor J.-S., Kennedy M.A. Solution-state structure of a DNA dodecamer duplex containing a Cis-Syn thymine cyclobutane dimer, the major UV photoproduct of DNA. J. Mol. Biol. 1998; 282:1013–1032. 9753551 10.1006/jmbi.1998.2062

[97] Fitch M.E., Nakajima S., Yasui A., Ford J.M. In vivo recruitment of XPC to UV-induced cyclobutane pyrimidine dimers by the DDB2 gene product. J. Biol. Chem. 2003; 278:46906–46910. 12944386 10.1074/jbc.M307254200

[98] Paul D., Mu H., Zhao H., Ouerfelli O., Jeffrey P.D., Broyde S., Min J.-H. Structure and mechanism of pyrimidine-pyrimidone (6-4) photoproduct recognition by the Rad4/XPC nucleotide excision repair complex. Nucleic Acids Res. 2019; 47:6015–6028. 31106376 10.1093/nar/gkz359 PMC6614856

[99] Gale J.M., Smerdon M.J. UV induced (6-4) photoproducts are distributed differently than cyclobutane dimers in nucleosomes. Photochem. Photobiol. 1990; 51:411–417. 2160660 10.1111/j.1751-1097.1990.tb01732.x

[100] Mitchell D.L., Nguyen T.D., Cleaver J.E. Nonrandom induction of pyrimidine-pyrimidone (6-4) photoproducts in ultraviolet-irradiated human chromatin. J. Biol. Chem. 1990; 265:5353–5356. 2318816

[101] Schul W., Jans J., Rijksen Y.M.A., Klemann K.H.M., Eker A.P.M., de Wit J., Nikaido O., Nakajima S., Yasui A., Hoeijmakers J.H.J. et al. Enhanced repair of cyclobutane pyrimidine dimers and improved UV resistance in photolyase transgenic mice. EMBO J. 2002; 21:4719–4729. 12198174 10.1093/emboj/cdf456 PMC125407

[102] Garinis G.A., Mitchell J.R., Moorhouse M.J., Hanada K., de Waard H., Vandeputte D., Jans J., Brand K., Smid M., van der Spek P.J. et al. Transcriptome analysis reveals cyclobutane pyrimidine dimers as a major source of UV-induced DNA breaks. EMBO J. 2005; 24:3952–3962. 16252008 10.1038/sj.emboj.7600849 PMC1283948

[103] Ikehata H., Ono T. The mechanisms of UV mutagenesis. J. Radiat. Res. 2011; 52:115–125. 21436607 10.1269/jrr.10175

[104] Scrima A., Koníčková R., Czyzewski B.K., Kawasaki Y., Jeffrey P.D., Groisman R., Nakatani Y., Iwai S., Pavletich N.P., Thomä N.H. Structural basis of UV DNA-damage recognition by the DDB1–DDB2 complex. Cell. 2008; 135:1213–1223. 19109893 10.1016/j.cell.2008.10.045 PMC2676164

[105] Fischer E.S., Scrima A., Böhm K., Matsumoto S., Lingaraju G.M., Faty M., Yasuda T., Cavadini S., Wakasugi M., Hanaoka F. et al. The molecular basis of CRL4(DDB2/CSA) ubiquitin ligase architecture, targeting, and activation. Cell. 2011; 147:1024–1039. 22118460 10.1016/j.cell.2011.10.035

[106] Sugasawa K. Molecular mechanisms of DNA damage recognition for mammalian nucleotide excision repair. DNA Repair (Amst). 2016; 44:110–117. 27264556 10.1016/j.dnarep.2016.05.015

[107] Zhu Q., Wani A.A. Nucleotide excision repair: finely tuned molecular orchestra of early pre-incision events. Photochem. Photobiol. 2017; 93:166–177. 27696486 10.1111/php.12647 PMC5315608

[108] Sugasawa K., Okuda Y., Saijo M., Nishi R., Matsuda N., Chu G., Mori T., Iwai S., Tanaka K., Tanaka K. et al. UV-induced ubiquitylation of XPC protein mediated by UV-DDB-ubiquitin ligase complex. Cell. 2005; 121:387–400. 15882621 10.1016/j.cell.2005.02.035

[109] Kapetanaki M.G., Guerrero-Santoro J., Bisi D.C., Hsieh C.L., Rapić-Otrin V., Levine A.S. The DDB1-CUL4A(DDB2) ubiquitin ligase is deficient in xeroderma pigmentosum group E and targets histone H2A at UV-damaged DNA sites. Proc. Natl. Acad. Sci. 2006; 103:2588–2593. 16473935 10.1073/pnas.0511160103 PMC1413840

[110] Wang H., Zhai L., Xu J., Joo H.-Y., Jackson S., Erdjument-Bromage H., Tempst P., Xiong Y., Zhang Y. Histone H3 and H4 ubiquitylation by the CUL4-DDB-ROC1 ubiquitin ligase facilitates cellular response to DNA damage. Mol. Cell. 2006; 22:383–394. 16678110 10.1016/j.molcel.2006.03.035

[111] Puumalainen M.-R., Lessel D., Rüthemann P., Kaczmarek N., Bachmann K., Ramadan K., Naegeli H. Chromatin retention of DNA damage sensors DDB2 and XPC through loss of p97 segregase causes genotoxicity. Nat. Commun. 2014; 5:3695. 24770583 10.1038/ncomms4695 PMC4007632

[112] He J., Zhu Q., Wani G., Sharma N., Han C., Qian J., Pentz K., Wang Q., Wani A.A. Ubiquitin-specific protease 7 regulates nucleotide excision repair through deubiquitinating XPC protein and preventing XPC protein from undergoing ultraviolet light-induced and VCP/p97 protein-regulated proteolysis. J. Biol. Chem. 2014; 289:27278–27289. 25118285 10.1074/jbc.M114.589812 PMC4175359

[113] El-Mahdy M.A., Zhu Q., Wang Q., Wani G., Praetorius-Ibba M., Wani A.A. Cullin 4A-mediated proteolysis of DDB2 protein at DNA damage sites regulates in vivo lesion recognition by XPC. J. Biol. Chem. 2006; 281:13404–13411. 16527807 10.1074/jbc.M511834200

[114] Chen X., Zhang Y., Douglas L., Zhou P. UV-damaged DNA-binding proteins are targets of CUL-4A-mediated ubiquitination and degradation. J. Biol. Chem. 2001; 276:48175–48182. 11673459 10.1074/jbc.M106808200

[115] Rapic-Otrin V., McLenigan M.P., Bisi D.C., Gonzalez M., Levine A.S. Sequential binding of UV DNA damage binding factor and degradation of the p48 subunit as early events after UV irradiation. Nucleic Acids Res. 2002; 30:2588–2598. 12034848 10.1093/nar/30.11.2588 PMC117178

[116] Poulsen S.L., Hansen R.K., Wagner S.A., van Cuijk L., van Belle G.J., Streicher W., Wikström M., Choudhary C., Houtsmuller A.B., Marteijn J.A. et al. RNF111/Arkadia is a SUMO-targeted ubiquitin ligase that facilitates the DNA damage response. J. Cell Biol. 2013; 201:797–807. 23751493 10.1083/jcb.201212075 PMC3678163

[117] van Cuijk L., van Belle G.J., Turkyilmaz Y., Poulsen S.L., Janssens R.C., Theil A.F., Sabatella M., Lans H., Mailand N., Houtsmuller A.B. et al. SUMO and ubiquitin-dependent XPC exchange drives nucleotide excision repair. Nat. Commun. 2015; 6:7499. 26151477 10.1038/ncomms8499 PMC4501428

[118] Akita M., Tak Y.-S., Shimura T., Matsumoto S., Okuda-Shimizu Y., Shimizu Y., Nishi R., Saitoh H., Iwai S., Mori T. et al. SUMOylation of xeroderma pigmentosum group C protein regulates DNA damage recognition during nucleotide excision repair. Sci. Rep. 2015; 5:10984. 26042670 10.1038/srep10984 PMC4455304

[119] Lan L., Nakajima S., Kapetanaki M.G., Hsieh C.L., Fagerburg M., Thickman K., Rodriguez-Collazo P., Leuba S.H., Levine A.S., Rapić-Otrin V. Monoubiquitinated histone H2A destabilizes photolesion-containing nucleosomes with concomitant release of UV-damaged DNA-binding protein E3 ligase. J. Biol. Chem. 2012; 287:12036–12049. 22334663 10.1074/jbc.M111.307058 PMC3320950

[120] Luijsterburg M.S., Lindh M., Acs K., Vrouwe M.G., Pines A., van Attikum H., Mullenders L.H., Dantuma N.P. DDB2 promotes chromatin decondensation at UV-induced DNA damage. J. Cell Biol. 2012; 197:267–281. 22492724 10.1083/jcb.201106074 PMC3328393

[121] Robu M., Shah R.G., Purohit N.K., Zhou P., Naegeli H., Shah G.M. Poly(ADP-ribose) polymerase 1 escorts XPC to UV-induced DNA lesions during nucleotide excision repair. Proc. Natl. Acad. Sci. U.S.A. 2017; 114:E6847–E6856. 28760956 10.1073/pnas.1706981114 PMC5565455

[122] Brand M., Moggs J.G., Oulad‐Abdelghani M., Lejeune F., Dilworth F.J., Stevenin J., Almouzni G., Tora L. UV-damaged DNA-binding protein in the TFTC complex links DNA damage recognition to nucleosome acetylation. EMBO J. 2001; 20:3187–3196. 11406595 10.1093/emboj/20.12.3187 PMC150203

[123] Datta A., Bagchi S., Nag A., Shiyanov P., Adami G.R., Yoon T., Raychaudhuri P. The p48 subunit of the damaged-DNA binding protein DDB associates with the CBP/p300 family of histone acetyltransferase. Mutat. Res. Repair. 2001; 486:89–97. 10.1016/s0921-8777(01)00082-9 11425514

[124] Cazzalini O., Perucca P., Savio M., Necchi D., Bianchi L., Stivala L.A., Ducommun B., Scovassi A.I., Prosperi E. Interaction of p21 CDKN1A with PCNA regulates the histone acetyltransferase activity of p300 in nucleotide excision repair. Nucleic Acids Res. 2008; 36:1713–1722. 18263614 10.1093/nar/gkn014 PMC2275133

[125] Guo R., Chen J., Mitchell D.L., Johnson D.G. GCN5 and E2F1 stimulate nucleotide excision repair by promoting H3K9 acetylation at sites of damage. Nucleic Acids Res. 2011; 39:1390–1397. 20972224 10.1093/nar/gkq983 PMC3045616

[126] Horikoshi N., Tachiwana H., Kagawa W., Osakabe A., Matsumoto S., Iwai S., Sugasawa K., Kurumizaka H. Crystal structure of the nucleosome containing ultraviolet light-induced cyclobutane pyrimidine dimer. Biochem. Biophys. Res. Commun. 2016; 471:117–122. 26837048 10.1016/j.bbrc.2016.01.170

[127] Cai Y., Fu I., Geacintov N.E., Zhang Y., Broyde S. Synergistic effects of H3 and H4 nucleosome tails on structure and dynamics of a lesion-containing DNA: Binding of a displaced lesion partner base to the H3 tail for GG-NER recognition. DNA Repair (Amst). 2018; 65:73–78. 29631253 10.1016/j.dnarep.2018.02.009 PMC5911426

[128] Otrin V.R., McLenigan M., Takao M., Levine A.S., Protic M. Translocation of a UV-damaged DNA binding protein into a tight association with chromatin after treatment of mammalian cells with UV light. J. Cell Sci. 1997; 110:1159–1168. 9191040 10.1242/jcs.110.10.1159

[129] Groisman R., Polanowska J., Kuraoka I., Sawada J., Saijo M., Drapkin R., Kisselev A.F., Tanaka K., Nakatani Y. The ubiquitin ligase activity in the DDB2 and CSA complexes is differentially regulated by the COP9 signalosome in response to DNA damage. Cell. 2003; 113:357–367. 12732143 10.1016/s0092-8674(03)00316-7

[130] Yasuda T., Sugasawa K., Shimizu Y., Iwai S., Shiomi T., Hanaoka F. Nucleosomal structure of undamaged DNA regions suppresses the non-specific DNA binding of the XPC complex. DNA Repair (Amst). 2005; 4:389–395. 15661662 10.1016/j.dnarep.2004.10.008

[131] Hoogstraten D., Bergink S., Ng J.M.Y., M Verbiest V.H., Luijsterburg M.S., Geverts B., Raams A., Dinant C., Hoeijmakers J.H., Vermeulen W. et al. Versatile DNA damage detection by the global genome nucleotide excision repair protein XPC. J. Cell Sci. 2008; 121:2850–2859. 18682493 10.1242/jcs.031708

[132] Solimando L., Luijsterburg M.S., Vecchio L., Vermeulen W., van Driel R., Fakan S. Spatial organization of nucleotide excision repair proteins after UV-induced DNA damage in the human cell nucleus. J. Cell Sci. 2009; 122:83–91. 19066286 10.1242/jcs.031062

[133] Fei J., Kaczmarek N., Luch A., Glas A., Carell T., Naegeli H. Regulation of nucleotide excision repair by UV-DDB: prioritization of damage recognition to internucleosomal DNA. PLoS Biol. 2011; 9:e1001183. 22039351 10.1371/journal.pbio.1001183 PMC3201922

[134] Balbo Pogliano C., Gatti M., Rüthemann P., Garajovà Z., Penengo L., Naegeli H. ASH1L histone methyltransferase regulates the handoff between damage recognition factors in global-genome nucleotide excision repair. Nat. Commun. 2017; 8:1333. 29109511 10.1038/s41467-017-01080-8 PMC5673894

[135] Gong F., Fahy D., Smerdon M.J. Rad4-Rad23 interaction with SWI/SNF links ATP-dependent chromatin remodeling with nucleotide excision repair. Nat. Struct. Mol. Biol. 2006; 13:902–907. 17013386 10.1038/nsmb1152

[136] Ray A., Mir S.N., Wani G., Zhao Q., Battu A., Zhu Q., Wang Q.-E., Wani A.A. Human SNF5/INI1, a component of the human SWI/SNF chromatin remodeling complex, promotes nucleotide excision repair by influencing ATM recruitment and downstream H2AX phosphorylation. Mol. Cell Biol. 2009; 29:6206–6219. 19805520 10.1128/MCB.00503-09 PMC2786693

[137] Peterson C.L., Almouzni G. Nucleosome dynamics as modular systems that integrate DNA damage and repair. Cold Spring Harb. Perspect. Biol. 2013; 5:a012658. 24003210 10.1101/cshperspect.a012658 PMC3753706

[138] Pines A., Vrouwe M.G., Marteijn J.A., Typas D., Luijsterburg M.S., Cansoy M., Hensbergen P., Deelder A., de Groot A., Matsumoto S. et al. PARP1 promotes nucleotide excision repair through DDB2 stabilization and recruitment of ALC1. J. Cell Biol. 2012; 199:235–249. 23045548 10.1083/jcb.201112132 PMC3471223

[139] Jiang Y., Wang X., Bao S., Guo R., Johnson D.G., Shen X., Li L. INO80 chromatin remodeling complex promotes the removal of UV lesions by the nucleotide excision repair pathway. Proc. Natl. Acad. Sci. U.S.A. 2010; 107:17274–17279. 20855601 10.1073/pnas.1008388107 PMC2951448

[140] Rüthemann P., Balbo Pogliano C., Codilupi T., Garajovà Z., Naegeli H. Chromatin remodeler CHD1 promotes XPC‐to‐TFIIH handover of nucleosomal UV lesions in nucleotide excision repair. EMBO J. 2017; 37:e201695742. 10.15252/embj.201695742 PMC5686551 29018037

[141] Skene P.J., Hernandez A.E., Groudine M., Henikoff S. The nucleosomal barrier to promoter escape by RNA polymerase II is overcome by the chromatin remodeler Chd1. Elife. 2014; 3:2042. 10.7554/eLife.02042 PMC3983905 24737864

[142] Zhao Q., Wang Q.-E., Ray A., Wani G., Han C., Milum K., Wani A.A. Modulation of nucleotide excision repair by mammalian SWI/SNF chromatin-remodeling complex. J. Biol. Chem. 2009; 284:30424–30432. 19740755 10.1074/jbc.M109.044982 PMC2781597

[143] Zhang L., Zhang Q., Jones K., Patel M., Gong F. The chromatin remodeling factor BRG1 stimulates nucleotide excision repair by facilitating recruitment of XPC to sites of DNA damage. Cell Cycle. 2009; 8:3953–3959. 19901545 10.4161/cc.8.23.10115

[144] Kouzarides T. Chromatin modifications and their function. Cell. 2007; 128:693–705. 17320507 10.1016/j.cell.2007.02.005

[145] Campos E.I., Reinberg D. Histones: annotating chromatin. Annu. Rev. Genet. 2009; 43:559–599. 19886812 10.1146/annurev.genet.032608.103928

[146] Wagner E.J., Carpenter P.B. Understanding the language of Lys36 methylation at histone H3. Nat. Rev. Mol. Cell Biol. 2012; 13:115–126. 22266761 10.1038/nrm3274 PMC3969746

[147] Varier R.A., Timmers H.T.M. Histone lysine methylation and demethylation pathways in cancer. Biochim. Biophys. Acta - Rev. Cancer. 2011; 1815:75–89. 10.1016/j.bbcan.2010.10.002 20951770

[148] Greer E.L., Shi Y. Histone methylation: a dynamic mark in health, disease and inheritance. Nat. Rev. Genet. 2012; 13:343–357. 22473383 10.1038/nrg3173 PMC4073795

[149] Zentner G.E., Henikoff S. Regulation of nucleosome dynamics by histone modifications. Nat. Struct. Mol. Biol. 2013; 20:259–266. 23463310 10.1038/nsmb.2470

[150] Black J.C., Van Rechem C., Whetstine J.R. Histone lysine methylation dynamics: establishment, regulation, and biological impact. Mol. Cell. 2012; 48:491–507. 23200123 10.1016/j.molcel.2012.11.006 PMC3861058

[151] Palomera-Sanchez Z., Bucio-Mendez A., Valadez-Graham V., Reynaud E., Zurita M. Drosophila p53 is required to increase the levels of the dKDM4B demethylase after UV-induced DNA damage to demethylate histone H3 lysine 9. J. Biol. Chem. 2010; 285:31370–31379. 20675387 10.1074/jbc.M110.128462 PMC2951211

[152] Castelli G., Pelosi E., Testa U. Targeting histone methyltransferase and demethylase in acute myeloid leukemia therapy. Onco. Targets. Ther. 2018; 11:131–155. 29343972 10.2147/OTT.S145971 PMC5749389

[153] Carlson S.M., Gozani O. Nonhistone lysine methylation in the regulation of cancer pathways. Cold Spring Harb. Perspect. Med. 2016; 6:a026435. 27580749 10.1101/cshperspect.a026435 PMC5088510

[154] Alam H., Gu B., Lee M.G. Histone methylation modifiers in cellular signaling pathways. Cell Mol. Life Sci. 2015; 72:4577–4592. 26305020 10.1007/s00018-015-2023-y PMC4628846

[155] Kudithipudi S., Jeltsch A. Approaches and guidelines for the identification of novel substrates of protein lysine methyltransferases. Cell Chem. Biol. 2016; 23:1049–1055. 27569752 10.1016/j.chembiol.2016.07.013

[156] Nguyen A.T., Zhang Y. The diverse functions of Dot1 and H3K79 methylation. Genes Dev. 2011; 25:1345–1358. 21724828 10.1101/gad.2057811 PMC3134078

[157] Vlaming H., van Leeuwen F. The upstreams and downstreams of H3K79 methylation by DOT1L. Chromosoma. 2016; 125:593–605. 26728620 10.1007/s00412-015-0570-5

[158] Game J.C., Williamson M.S., Spicakova T., Brown J.M. The RAD6/BRE1 histone modification pathway in Saccharomyces confers radiation resistance through a RAD51-dependent process that is independent of RAD18. Genetics. 2006; 173:1951–1968. 16783014 10.1534/genetics.106.057794 PMC1569736

[159] Nakanishi S., Lee J.S., Gardner K.E., Gardner J.M., Takahashi Y., Chandrasekharan M.B., Sun Z.-W., Osley M.A., Strahl B.D., Jaspersen S.L. et al. Histone H2BK123 monoubiquitination is the critical determinant for H3K4 and H3K79 trimethylation by COMPASS and Dot1. J. Cell Biol. 2009; 186:371–377. 19667127 10.1083/jcb.200906005 PMC2728409

[160] Ng H.H., Xu R.-M., Zhang Y., Struhl K. Ubiquitination of histone H2B by Rad6 is required for efficient Dot1-mediated methylation of histone H3 lysine 79. J. Biol. Chem. 2002; 277:34655–34657. 12167634 10.1074/jbc.C200433200

[161] McGinty R.K., Kim J., Chatterjee C., Roeder R.G., Muir T.W. Chemically ubiquitylated histone H2B stimulates hDot1L-mediated intranucleosomal methylation. Nature. 2008; 453:812–816. 18449190 10.1038/nature06906 PMC3774535

[162] Huyen Y., Zgheib O., DiTullio R.A. Jr, Gorgoulis V.G., Zacharatos P., Petty T.J., Sheston E.A., Mellert H.S., Stavridi E.S., Halazonetis T.D. Methylated lysine 79 of histone H3 targets 53BP1 to DNA double-strand breaks. Nature. 2004; 432:406–411. 15525939 10.1038/nature03114

[163] Singer M.S., Kahana A., Wolf A.J., Meisinger L.L., Peterson S.E., Goggin C., Mahowald M., Gottschling D.E. Identification of high-copy disruptors of telomeric silencing in Saccharomyces cerevisiae. Genetics. 1998; 150:613–632. 9755194 10.1093/genetics/150.2.613 PMC1460361

[164] Wang Z., Zang C., Rosenfeld J.A., Schones D.E., Barski A., Cuddapah S., Cui K., Roh T.-Y., Peng W., Zhang M.Q. et al. Combinatorial patterns of histone acetylations and methylations in the human genome. Nat. Genet. 2008; 40:897–903. 18552846 10.1038/ng.154 PMC2769248

[165] Steger D.J., Lefterova M.I., Ying L., Stonestrom A.J., Schupp M., Zhuo D., Vakoc A.L., Kim J.-E., Chen J., Lazar M.A. et al. DOT1L/KMT4 recruitment and H3K79 methylation are ubiquitously coupled with gene transcription in mammalian cells. Mol. Cell. Biol. 2008; 28:2825–2839. 18285465 10.1128/MCB.02076-07 PMC2293113

[166] Jonkers I., Kwak H., Lis J.T. Genome-wide dynamics of Pol II elongation and its interplay with promoter proximal pausing, chromatin, and exons. Elife. 2014; 3:e02407. 24843027 10.7554/eLife.02407 PMC4001325

[167] Veloso A., Kirkconnell K.S., Magnuson B., Biewen B., Paulsen M.T., Wilson T.E., Ljungman M. Rate of elongation by RNA polymerase II is associated with specific gene features and epigenetic modifications. Genome Res. 2014; 24:896–905. 24714810 10.1101/gr.171405.113 PMC4032854

[168] Wood K., Tellier M., Murphy S., Wood K., Tellier M., Murphy S. DOT1L and H3K79 methylation in transcription and genomic stability. Biomolecules. 2018; 8:11. 10.3390/biom8010011 PMC5871980 29495487

[169] Godfrey L., Crump N.T., Thorne R., Lau I.-J., Repapi E., Dimou D., Smith A.L., Harman J.R., Telenius J.M., Marieke Oudelaar A. et al. DOT1L inhibition reveals a distinct subset of enhancers dependent on H3K79 methylation. Nat. Commun. 2019; 10:2803. 31243293 10.1038/s41467-019-10844-3 PMC6594956

[170] Wysocki R., Javaheri A., Allard S., Sha F., Côté J., Kron S.J. Role of Dot1-dependent histone H3 methylation in G1 and S phase DNA damage checkpoint functions of Rad9. Mol. Cell Biol. 2005; 25:8430–8443. 16166626 10.1128/MCB.25.19.8430-8443.2005 PMC1265753

[171] Giannattasio M., Lazzaro F., Plevani P., Muzi-Falconi M. The DNA damage checkpoint response requires histone H2B ubiquitination by Rad6-Bre1 and H3 methylation by Dot1. J. Biol. Chem. 2005; 280:9879–9886. 15632126 10.1074/jbc.M414453200

[172] Bostelman L.J., Keller A.M., Albrecht A.M., Arat A., Thompson J.S. Methylation of histone H3 lysine-79 by Dot1p plays multiple roles in the response to UV damage in Saccharomyces cerevisiae. DNA Repair (Amst). 2007; 6:383–395. 17267293 10.1016/j.dnarep.2006.12.010

[173] Chaudhuri S., Wyrick J.J., Smerdon M.J. Histone H3 Lys79 methylation is required for efficient nucleotide excision repair in a silenced locus of Saccharomyces cerevisiae. Nucleic Acids Res. 2009; 37:1690–1700. 19155276 10.1093/nar/gkp003 PMC2655692

[174] Müller-Taubenberger A., Bönisch C., Fürbringer M., Wittek F., Hake S.B. The histone methyltransferase Dot1 is required for DNA damage repair and proper development in Dictyostelium. Biochem. Biophys. Res. Commun. 2011; 404:1016–1022. 21187070 10.1016/j.bbrc.2010.12.101

[175] Tatum D., Li S. Evidence that the histone methyltransferase Dot1 mediates global genomic repair by methylating histone H3 on lysine 79. J. Biol. Chem. 2011; 286:17530–17535. 21460225 10.1074/jbc.M111.241570 PMC3093827

[176] Jones B., Su H., Bhat A., Lei H., Bajko J., Hevi S., Baltus G.A., Kadam S., Zhai H., Valdez R. et al. The histone H3K79 methyltransferase Dot1L is essential for mammalian development and heterochromatin structure. PLoS Genet. 2008; 4:e1000190. 18787701 10.1371/journal.pgen.1000190 PMC2527135

[177] Feng Y., Yang Y., Ortega M.M., Copeland J.N., Zhang M., Jacob J.B., Fields T.A., Vivian J.L., Fields P.E. Early mammalian erythropoiesis requires the Dot1L methyltransferase. Blood. 2010; 116:4483–4491. 20798234 10.1182/blood-2010-03-276501 PMC3321834

[178] Jo S.Y., Granowicz E.M., Maillard I., Thomas D., Hess J.L. Requirement for Dot1l in murine postnatal hematopoiesis and leukemogenesis by MLL translocation. Blood. 2011; 117:4759–4768. 21398221 10.1182/blood-2010-12-327668 PMC3100687

[179] Zhu B., Chen S., Wang H., Yin C., Han C., Peng C., Liu Z., Wan L., Zhang X., Zhang J. et al. The protective role of DOT1L in UV-induced melanomagenesis. Nat. Commun. 2018; 9:259. 29343685 10.1038/s41467-017-02687-7 PMC5772495

[180] Oksenych V., Zhovmer A., Ziani S., Mari P.-O., Eberova J., Nardo T., Stefanini M., Giglia-Mari G., Egly J.-M., Coin F. Histone methyltransferase DOT1L drives recovery of gene expression after a genotoxic attack. PLoS Genet. 2013; 9:e1003611. 23861670 10.1371/journal.pgen.1003611 PMC3701700

[181] Sutherland B.M., Hacham H., Bennett P., Sutherland J.C., Moran M., Gange R.W. Repair of cyclobutyl pyrimidine dimers in human skin: Variability among normal humans in nucleotide excision and in photorepair. Photodermatol. Photoimmunol. Photomed. 2002; 18:109–116. 12207672 10.1034/j.1600-0781.2002.00748.x

[182] Hwang B.J., Ford J.M., Hanawalt P.C., Chu G. Expression of the p48 xeroderma pigmentosum gene is p53-dependent and is involved in global genomic repair. Proc. Natl. Acad. Sci. U.S.A. 1999; 96:424–428. 9892649 10.1073/pnas.96.2.424 PMC15152

[183] Huang C., Zhu B. Roles of H3K36-specific histone methyltransferases in transcription: antagonizing silencing and safeguarding transcription fidelity. Biophys. Reports. 2018; 4:170–177. 10.1007/s41048-018-0063-1 PMC6153486 30310854

[184] Shearn A. The ash-1, ash-2 and trithorax genes of Drosophila melanogaster are functionally related. Genetics. 1989; 121:517–525. 2497049 10.1093/genetics/121.3.517 PMC1203637

[185] Tripoulas N.A., Hersperger E., La Jeunesse D., Shearn A. Molecular genetic analysis of the Drosophila melanogaster gene absent, small or homeotic discs1 (ash1). Genetics. 1994; 137:1027–1038. 7982557 10.1093/genetics/137.4.1027 PMC1206050

[186] Dorighi K.M., Tamkun J.W. The trithorax group proteins Kismet and ASH1 promote H3K36 dimethylation to counteract Polycomb group repression in Drosophila. Dev. 2013; 140:4182–4192. 10.1242/dev.095786 PMC3787758 24004944

[187] Klymenko T., Müller J. The histone methyltransferases Trithorax and Ash1 prevent transcriptional silencing by Polycomb group proteins. EMBO Rep. 2004; 5:373–377. 15031712 10.1038/sj.embor.7400111 PMC1299022

[188] An S., Joo Yeo K., Ho Jeon Y., Song J.-J. Crystal structure of the human histone methyltransferase ASH1L catalytic domain and its implications for the regulatory mechanism. J. Biol. Chem. 2011; 286:8369–8374. 21239497 10.1074/jbc.M110.203380 PMC3048721

[189] Hou P., Huang C., Liu C.-P., Yang N., Yu T., Yin Y., Zhu B., Xu R.-M. Structural insights into stimulation of Ash1L’s H3K36 methyltransferase activity through Mrg15 binding. Structure. 2019; 27:837–845. 30827843 10.1016/j.str.2019.01.015

[190] Lee Y., Yoon E., Cho S., Schmähling S., Müller J., Song J.-J. Structural basis of MRG15-mediated activation of the ASH1L histone methyltransferase by releasing an autoinhibitory loop. Structure. 2019; 27:846–852. 30827841 10.1016/j.str.2019.01.016

[191] Tanaka Y., Katagiri Z., Kawahashi K., Kioussis D., Kitajima S. Trithorax-group protein ASH1 methylates histone H3 lysine 36. Gene. 2007; 397:161–168. 17544230 10.1016/j.gene.2007.04.027

[192] Yuan G., Ma B., Yuan W., Zhang Z., Chen P., Ding X., Feng L., Shen X., Chen S., Li G. et al. Histone H2A ubiquitination inhibits the enzymatic activity of H3 lysine 36 methyltransferases. J. Biol. Chem. 2013; 288:30832–30842. 24019522 10.1074/jbc.M113.475996 PMC3829398

[193] Miyazaki H., Higashimoto K., Yada Y., Endo T.A., Sharif J., Komori T., Matsuda M., Koseki Y., Nakayama M., Soejima H. et al. Ash1l methylates Lys36 of histone H3 independently of transcriptional elongation to counteract Polycomb silencing. PLoS Genet. 2013; 9:e1003897. 24244179 10.1371/journal.pgen.1003897 PMC3820749

[194] Eram M.S., Kuznetsova E., Li F., Lima-Fernandes E., Kennedy S., Chau I., Arrowsmith C.H., Schapira M., Vedadi M. Kinetic characterization of human histone H3 lysine 36 methyltransferases, ASH1L and SETD2. Biochim. Biophys. Acta - Gen. Subj. 2015; 1850:1842–1848. 10.1016/j.bbagen.2015.05.013 26002201

[195] Gregory G.D., Vakoc C.R., Rozovskaia T., Zheng X., Patel S., Nakamura T., Canaani E., Blobel G.A. Mammalian ASH1L is a histone methyltransferase that occupies the transcribed region of active genes. Mol. Cell Biol. 2007; 27:8466–8479. 17923682 10.1128/MCB.00993-07 PMC2169421

[196] Yin B., Yu F., Wang C., Li B., Liu M., Ye L. Epigenetic control of mesenchymal stem cell fate decision via histone methyltransferase Ash1l. Stem Cells. 2019; 37:115–127. 30270478 10.1002/stem.2918

[197] Zhu T., Liang C., Li D., Tian M., Liu S., Gao G., Guan J.-S. Histone methyltransferase Ash1L mediates activity-dependent repression of neurexin-1α. Sci. Rep. 2016; 6:26597. 27229316 10.1038/srep26597 PMC4882582

[198] Shen W., Krautscheid P., Rutz A.M., Bayrak-Toydemir P., Dugan S.L. De novo loss-of-function variants of ASH1L are associated with an emergent neurodevelopmental disorder. Eur. J. Med. Genet. 2018; 62:55–60. 29753921 10.1016/j.ejmg.2018.05.003

[199] Faundes V., Newman W.G., Bernardini L., Canham N., Clayton-Smith J., Dallapiccola B., Davies S.J., Demos M.K., Goldman A., Gill H. et al. Histone lysine methylases and demethylases in the landscape of human developmental disorders. Am. J. Hum. Genet. 2018; 102:175–187. 29276005 10.1016/j.ajhg.2017.11.013 PMC5778085

[200] Brinkmeier M.L., Geister K.A., Jones M., Waqas M., Maillard I., Camper S.A. The histone methyltransferase gene Absent, small, or homeotic discs-1 like is required for normal hox gene expression and fertility in mice. Biol. Reprod. 2015; 93:121. 26333994 10.1095/biolreprod.115.131516 PMC4712006

[201] Jones M., Chase J., Brinkmeier M., Xu J., Weinberg D.N., Schira J., Friedman A., Malek S., Grembecka J., Cierpicki T. et al. Ash1l controls quiescence and self-renewal potential in hematopoietic stem cells. J. Clin. Invest. 2015; 125:2007–2020. 25866973 10.1172/JCI78124 PMC4463197

[202] Nakamura T., Blechman J., Tada S., Rozovskaia T., Itoyama T., Bullrich F., Mazo A., Croce C.M., Geiger B., Canaani E. huASH1 protein, a putative transcription factor encoded by a human homologue of the Drosophila ash1 gene, localizes to both nuclei and cell-cell tight junctions. Proc. Natl. Acad. Sci. U.S.A. 2000; 97:7284–7289. 10860993 10.1073/pnas.97.13.7284 PMC16537

[203] Li G., Ye Z., Shi C., Sun L., Han M., Zhuang Y., Xu T., Zhao S., Wu X. The histone methyltransferase Ash1l is required for epidermal homeostasis in mice. Sci. Rep. 2017; 7:45401. 28374742 10.1038/srep45401 PMC5379632

[204] Song Y., Li L., Ou Y., Gao Z., Li E., Li X., Zhang W., Wang J.J., Xu L., Zhou Y. et al. Identification of genomic alterations in oesophageal squamous cell cancer. Nature. 2014; 509:91–95. 24670651 10.1038/nature13176

[205] Liu L., Kimball S., Liu H., Holowatyj A., Yang Z.-Q. Genetic alterations of histone lysine methyltransferases and their significance in breast cancer. Oncotarget. 2015; 6:2466–2482. 25537518 10.18632/oncotarget.2967 PMC4385864

[206] Fujimoto A., Furuta M., Totoki Y., Tsunoda T., Kato M., Shiraishi Y., Tanaka H., Taniguchi H., Kawakami Y., Ueno M. et al. Whole-genome mutational landscape and characterization of noncoding and structural mutations in liver cancer. Nat. Genet. 2016; 48:500–509. 27064257 10.1038/ng.3547

[207] Zhang Y., Wu W., Qu H. Integrated analysis of the gene expression changes during colorectal cancer progression by bioinformatic methods. J. Comput. Biol. 2019; 26:1168–1176. 31246501 10.1089/cmb.2019.0056

[208] Chesi M., Nardini E., Lim R.S.C., Smith K.D., Kuehl W.M., Bergsagel P.L. The t(4;14) translocation in myeloma dysregulates both FGFR3 and a novel gene, MMSET, resulting in IgH/MMSET hybrid transcripts. Blood. 1998; 9:3025–3034. 9787135

[209] Finelli P., Fabris S., Zagano S., Baldini L., Intini D., Nobili L., Lombardi L., Maiolo A.T., Neri A. Detection of t(4;14)(p16.3;q32) chromosomal translocation in multiple myeloma by double-color fluorescent in situ hybridization. Blood. 1999; 94:724–732. 10397739

[210] Stec I., Wright T.J., van Ommen G.-J.B., de Boer P.A.J., van Haeringen A., Moorman A.F.M., Altherr M.R., den Dunnen J.T. WHSC1, a 90 kb SET domain-containing gene, expressed in early development and homologous to a Drosophila dysmorphy gene maps in the Wolf-Hirschhorn syndrome critical region and is fused to IgH in t(1;14) multiple myeloma. Hum. Mol. Genet. 1998; 7:1071–1082. 9618163 10.1093/hmg/7.7.1071

[211] Bergemann A.D., Cole F., Hirschhorn K. The etiology of Wolf–Hirschhorn syndrome. Trends Genet. 2005; 21:188–195. 15734578 10.1016/j.tig.2005.01.008

[212] Hudlebusch H.R., Santoni-Rugiu E., Simon R., Ralfkiaer E., Rossing H.H., Johansen J. V., Jorgensen M., Sauter G., Helin K. The histone methyltransferase and putative oncoprotein MMSET is overexpressed in a large variety of human tumors. Clin. Cancer Res. 2011; 17:2919–2933. 21385930 10.1158/1078-0432.CCR-10-1302

[213] Asangani I.A., Ateeq B., Cao Q., Dodson L., Pandhi M., Kunju L.P., Mehra R., Lonigro R.J., Siddiqui J., Palanisamy N. et al. Characterization of the EZH2-MMSET histone methyltransferase regulatory axis in cancer. Mol. Cell. 2013; 49:80–93. 23159737 10.1016/j.molcel.2012.10.008 PMC3547524

[214] Nimura K., Ura K., Shiratori H., Ikawa M., Okabe M., Schwartz R.J., Kaneda Y. A histone H3 lysine 36 trimethyltransferase links Nkx2-5 to Wolf–Hirschhorn syndrome. Nature. 2009; 460:287–291. 19483677 10.1038/nature08086

[215] Pei H., Zhang L., Luo K., Qin Y., Chesi M., Fei F., Bergsagel P.L., Wang L., You Z., Lou Z. MMSET regulates histone H4K20 methylation and 53BP1 accumulation at DNA damage sites. Nature. 2011; 470:124–128. 21293379 10.1038/nature09658 PMC3064261

[216] Pei H., Wu X., Liu T., Yu K., Jelinek D.F., Lou Z. The histone methyltransferase MMSET regulates class switch recombination. J. Immunol. 2013; 190:756–763. 23241889 10.4049/jimmunol.1201811 PMC3775478

[217] Sarai N., Nimura K., Tamura T., Kanno T., Patel M.C., Heightman T.D., Ura K., Ozato K. WHSC1 links transcription elongation to HIRA-mediated histone H3.3 deposition. EMBO J. 2013; 32:2392–2406. 23921552 10.1038/emboj.2013.176 PMC3770338

[218] Chitale S., Richly H. DICER and ZRF1 contribute to chromatin decondensation during nucleotide excision repair. Nucleic Acids Res. 2017; 45:5901–5912. 28402505 10.1093/nar/gkx261 PMC5449631

[219] Chitale S., Richly H. DICER- and MMSET-catalyzed H4K20me2 recruits the nucleotide excision repair factor XPA to DNA damage sites. J. Cell Biol. 2018; 217:527–540. 29233865 10.1083/jcb.201704028 PMC5800799

[220] Hartlerode A.J., Guan Y., Rajendran A., Ura K., Schotta G., Xie A., Shah J. V., Scully R. Impact of histone H4 lysine 20 methylation on 53BP1 responses to chromosomal double strand breaks. PLoS One. 2012; 7:e49211. 23209566 10.1371/journal.pone.0049211 PMC3509127

[221] Sanders S.L., Portoso M., Mata J., Bähler J., Allshire R.C., Kouzarides T. Methylation of histone H4 lysine 20 controls recruitment of Crb2 to sites of DNA damage. Cell. 2004; 119:603–614. 15550243 10.1016/j.cell.2004.11.009

[222] Schotta G., Sengupta R., Kubicek S., Malin S., Kauer M., Callén E., Celeste A., Pagani M., Opravil S., De La Rosa-Velazquez I.A. et al. A chromatin-wide transition to H4K20 monomethylation impairs genome integrity and programmed DNA rearrangements in the mouse. Genes Dev. 2008; 22:2048–2061. 18676810 10.1101/gad.476008 PMC2492754

[223] Bennett R.L., Swaroop A., Troche C., Licht J.D. The role of nuclear receptor-binding SET domain family histone lysine methyltransferases in cancer. Cold Spring Harb. Perspect. Med. 2017; 7:a026708. 28193767 10.1101/cshperspect.a026708 PMC5453381

[224] Hake S.B., Garcia B.A., Duncan E.M., Kauer M., Dellaire G., Shabanowitz J., Bazett-Jones D.P., Allis C.D., Hunt D.F. Expression patterns and post-translational modifications associated with mammalian histone H3 variants. J. Biol. Chem. 2006; 281:559–568. 16267050 10.1074/jbc.M509266200

[225] Jang S., Kang C., Yang H.-S., Jung T., Hebert H., Chung K.Y., Kim S.J., Hohng S., Song J.-J. Structural basis of recognition and destabilization of the histone H2B ubiquitinated nucleosome by the DOT1L histone H3 Lys79 methyltransferase. Genes Dev. 2019; 33:620–625. 30923167 10.1101/gad.323790.118 PMC6546062

[226] Worden E.J., Hoffmann N.A., Hicks C.W., Wolberger C. Mechanism of cross-talk between H2B ubiquitination and H3 methylation by Dot1L. Cell. 2019; 176:1490–1501. 30765112 10.1016/j.cell.2019.02.002 PMC6498860

[227] Oh S., Jeong K., Kim H., Kwon C.S., Lee D. A lysine-rich region in Dot1p is crucial for direct interaction with H2B ubiquitylation and high level methylation of H3K79. Biochem. Biophys. Res. Commun. 2010; 399:512–517. 20678485 10.1016/j.bbrc.2010.07.100

[228] Stoddard C.I., Feng S., Campbell M.G., Liu W., Wang H., Zhong X., Bernatavichute Y., Cheng Y., Jacobsen S.E., Narlikar G.J. A nucleosome bridging mechanism for activation of a maintenance DNA methyltransferase. Mol. Cell. 2019; 73:73–83. 30415948 10.1016/j.molcel.2018.10.006 PMC6407616

[229] Kang H.-B., Choi Y., Lee J.M., Choi K.-C., Kim H.-C., Yoo J.-Y., Lee Y.-H., Yoon H.-G. The histone methyltransferase, NSD2, enhances androgen receptor-mediated transcription. FEBS Lett. 2009; 583:1880–1886. 19481544 10.1016/j.febslet.2009.05.038

